# The Athabasca River regulates methylmercury burdens of waterbirds breeding downstream

**DOI:** 10.1038/s41598-026-35970-z

**Published:** 2026-01-17

**Authors:** John Chételat, Craig Hebert, Jason D. Demers, Colin A. Cooke, Christine McClelland, Maureen Angell, Bridgit Bergquist, Marlene Evans, Maria F. Fahnestock, Kuzey Güneşli, Sarah Greenwood, Bruce Maclean, Mark McMaster, Lukas Mundy, Gerald Tetreault, Philippe J. Thomas

**Affiliations:** 1 Environment and Climate Change Canada, National Wildlife Research Centre, Ottawa, Ontario Canada; 2 Earth Systems Research Center, University of New Hampshire, Durham, NH USA; 3 Environment and Protected Areas, Government of Alberta, Edmonton, Alberta Canada; 4 Earth and Atmospheric Sciences, University of Alberta, Edmonton, Alberta Canada; 5 Department of Earth Sciences, University of Toronto, Toronto, Ontario Canada; 6 Environment and Climate Change Canada, National Hydrology Research Centre, Saskatoon, Saskatchewan Canada; 7 Joan and James Leitzel Center for Mathematics, Science, and Engineering Education, University of New Hampshire, Durham, NH USA; 8 Maclean Environmental Consulting, Winnipeg, Manitoba Canada; 9 Environment and Climate Change Canada, Canada Centre for Inland Waters, Burlington, Ontario Canada

**Keywords:** Mercury isotopes, River transport, High flow, Bioaccumulation, Terrestrial sources, Ecology, Ecology, Environmental sciences

## Abstract

This study characterized the transport pathways and sources of mercury in downstream food webs of the Athabasca River Basin in northern Alberta, Canada. Flowing through a large boreal watershed in the Athabasca Oil Sands Region, the river drains into the ecologically-sensitive Peace-Athabasca Delta and western Lake Athabasca. Mercury stable isotope measurements on abiotic and biotic matrices were combined with spatial and temporal sampling to evaluate the importance of the Athabasca River as a conduit for mercury. Mixing model estimates derived from mercury isotopes indicated the Athabasca River was the source of 62–94% of the bioaccumulated mercury in otter, fish, and tern eggs collected from the delta or lake. A time series from 2009 to 2022 showed mercury loads from the Athabasca River enhanced bioaccumulation in western Lake Athabasca with the doubling of total mercury (THg) concentration in eggs of breeding terns following high flow years compared to low flow years. Mercury isotope data from potential abiotic sources (Athabasca River and Lake sediment, leaf litter, soil, air, rain) suggested a predominately terrestrial origin of mercury in fish of the Athabasca River. The similarity of terrestrial mercury isotope signatures with oil sands-related matrices (natural bitumen seeps, industry samples) precluded an estimation of contributions from oil sands operations to mercury bioaccumulation. Catchment processes or disturbances that increase mercury loads in the Athabasca River will increase mercury concentrations of aquatic biota in downstream receiving waters.

**Supplementary Information:**

The online version contains supplementary material available at 10.1038/s41598-026-35970-z.

## Introduction

Rivers are important conduits of mercury, linking atmospheric deposition of this toxic metal on terrestrial surfaces with downstream aquatic environments. Large inland lakes receive substantial loads of mercury from tributaries, which contribute to mercury accumulation in those freshwater ecosystems^1,2^. On a global scale, rivers are the primary pathway delivering mercury to coastal oceans^3^. Rivers transport both inorganic mercury and methylmercury (MeHg) in different phases (particulate, dissolved, colloidal) related to various watershed sources and processes^4–6^. Watershed export of mercury is mediated by hydrology (with greater fluxes during high flows) via erosion of mineral and organic soil particles, resuspension of river sediments, and terrestrial runoff of mercury bound to dissolved organic matter^7–9^. Watershed features such as wetlands, vegetation type, land use, topography, and soil type play a critical role in determining the speciation, phase and flux of mercury^5,10^. While most of the total mercury (THg) exported from watersheds is inorganic, fluxes of MeHg from soils and wetlands can also be substantial, particularly in comparison to other potential MeHg sources in downstream receiving waters^2,11–13^. The impact of large river transport on MeHg bioaccumulation in downstream food webs remains challenging to evaluate because of complex processes related to hydrology, ecosystem mercury cycling (including mercury methylation), and food web dynamics^10,14–18^.

Mercury stable isotope analysis is a powerful tool to trace mercury sources, cycling, and transport processes in the environment^19^. Over the last two decades, the fractionation of mercury isotopes has been characterized in relation to different processes in the mercury cycle^19–23^, though some aspects such as fractionation during MeHg formation remain less well understood^24^. The mass dependent fractionation (MDF) of the ^202^Hg isotope and mass-independent fractionation (MIF) of odd isotopes (^199^Hg, ^201^Hg) and even isotopes (^200^Hg, ^204^Hg) result in environmental isotopic gradients that have been effective for evaluating deposition pathways and exposure pathways to food webs^25–27^. Trophic transfer does not result in MDF and MIF of mercury isotopes, making multi-isotope measurements on biota effective to distinguish: (1) mercury sources using δ^202^Hg^28,29^, (2) gradients of aqueous MeHg photodegradation prior to biological uptake using Δ^199^Hg^30,31^, and (3) contributions of wet Hg(II) deposition to bioaccumulation using Δ^200^Hg and Δ^204^Hg^15,32^.

Hebert^33^ used mercury isotopes to investigate drivers of bioaccumulation in the Athabasca River Basin, a vast boreal river-wetland-lake complex in northern Alberta (Canada). That investigation was initiated because an earlier spatial analysis revealed eggs of colonial waterbirds breeding in downstream receiving waters of the Athabasca River had higher THg concentrations than eggs of birds breeding in adjacent regions to the north and south^34^. Waterbird eggs are well-established bioindicators of local environmental conditions because the chemical composition of the eggs, including mercury, reflects the local diet of the female at the time of breeding^35–37^. Hebert^33^ showed larger inflows from the Athabasca River were correlated with higher THg concentrations in colonial waterbird eggs. Further, eggs collected after high flow years had less MIF of Δ^199^Hg, thereby potentially linking enhanced bioaccumulation in the eggs with river influence. However, a lack of mercury isotope data for other food web components and for potential mercury sources prevented a detailed characterization of river contributions to downstream bioaccumulation.

**Fig. 1:**
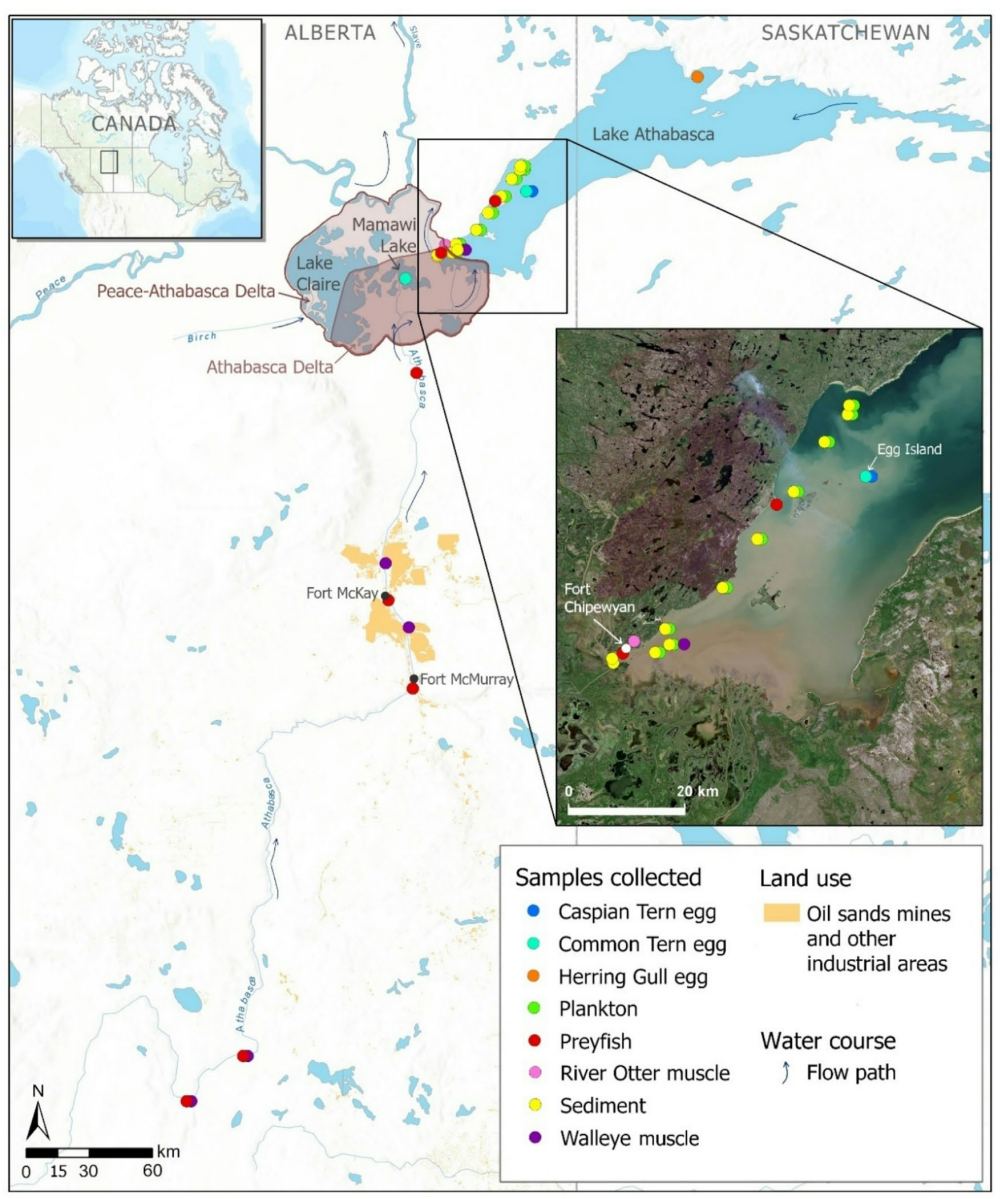
Study area and sampling locations in lower reaches of the Athabasca River Basin in northern Alberta, Canada. Approximate collection locations are displayed for River Otter in the Peace-Athabasca Delta and Walleye of Lake Athabasca. Map generated with ArcGIS Pro 3.2.2 (www.esri.com; mapping credits: Esri, TomTom, Garmin, FAO, NOAA, USGS, EPA, NRCan, Parks Canada, CGIAR, Canadian Community Maps contributors) and edited with GIMP 2.8.22 (www.gimp.org). The inset image of western Lake Athabasca was obtained courtesy of Sentinel-2 data (www.browser.dataspace.copernicus.eu) and taken on June 25, 2023, one month before the survey.

The importance of the Athabasca River as a conduit for mercury is of broad interest to local First Nations, Métis, governments, and industry because extensive surface mining of oil sands occurs adjacent to the river and ecologically-sensitive water bodies are located downstream (Fig. 1). The Athabasca River is the main source of water entering the Peace-Athabasca Delta (PAD) and western Lake Athabasca^38,39^, which support fish and wildlife that are traditional foods of local Indigenous Peoples. The PAD is listed by the United Nations as a heritage site of world importance under their Educational, Scientific and Cultural Organization (UNESCO) in part because of the critical aquatic habitat for migratory birds, including the endangered Whooping Crane (*Grus americana*)^38^. Mercury emitted from oil sands activities has been shown to deposit on the landscape near operations and may subsequently drain into the Athabasca River via runoff and tributaries^40–43^. Likewise, oil sands operations have accumulated 1.4 trillion L of toxic tailings pond water^44^, and the release of treated effluent into the Athabasca River is being considered as a future strategy to manage this wastewater.

Here we characterize in detail the transport and sources of mercury bioaccumulating in downstream receiving environments of the Athabasca River Basin. Building on earlier published work^33^, we generated new mercury isotope data for diverse aquatic biota (birds, fish, otter) covering a broader geographic area (Fig. 1), as well as for potential abiotic mercury sources (air, sediment, bitumen) in the basin. We evaluated the contributions of lake and river transport pathways for biota using a mercury isotope mixing model and accounted for the influence of food web structure using carbon and nitrogen stable isotopes^45^. With publicly available water chemistry data for the Athabasca River, we estimated THg and MeHg loads to downstream receiving waters. We conducted transect sampling of surface sediment and plankton to track the zone of river influence into western Lake Athabasca. Together, these multiple lines of evidence provided a robust analysis of mercury dynamics in the Athabasca River Basin. The findings give new insights into the complex processes linking mercury transport with food web exposure in river-influenced systems.

## Results

### Mercury stable isotopes of aquatic biota in the Athabasca River Basin

**Fig. 2:**
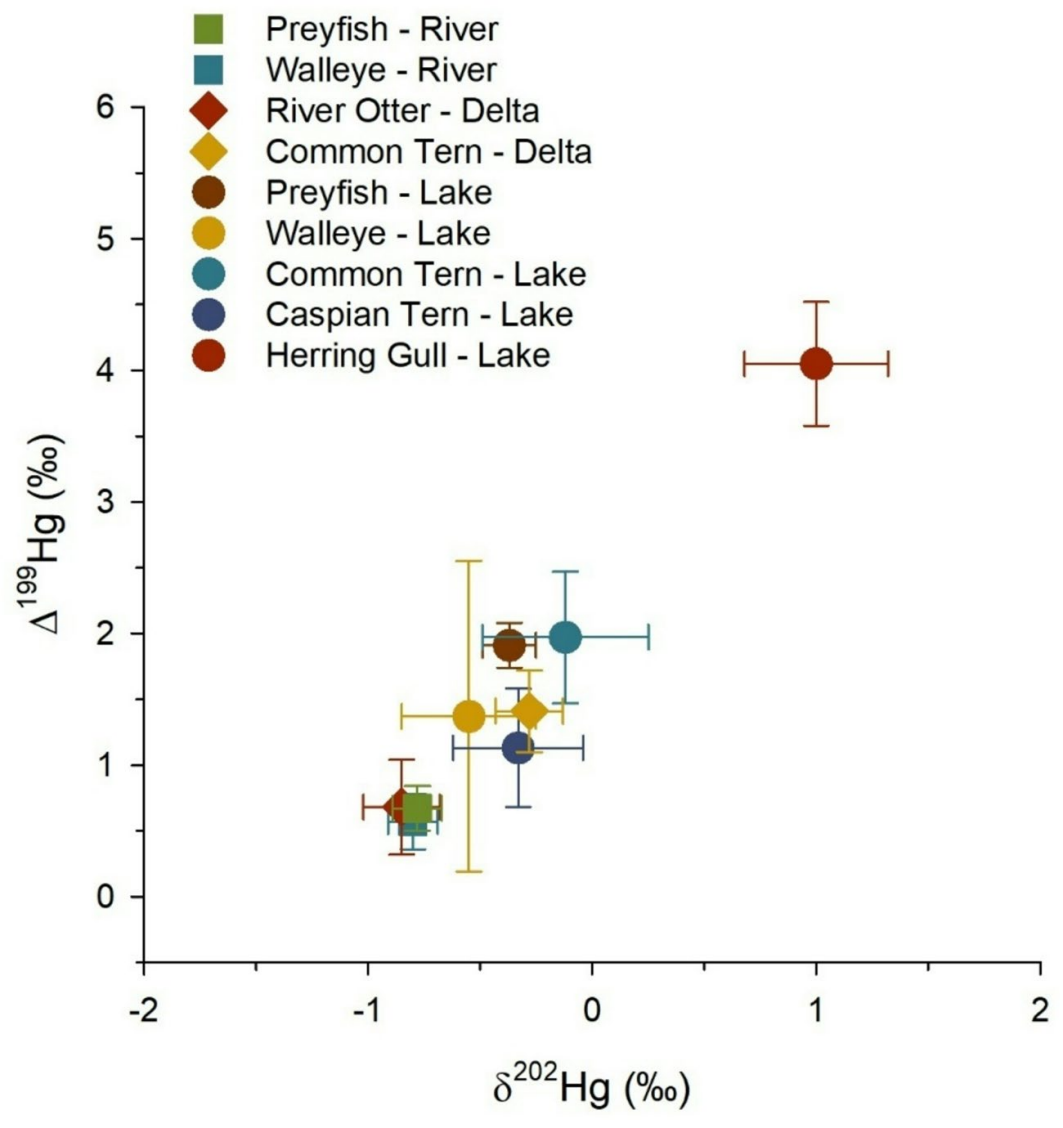
Mercury isotope biplot (δ^202^Hg, Δ^199^Hg) for aquatic biota in the Athabasca River, Peace-Athabasca Delta, and Lake Athabasca. Data points are means (± standard error) and sample sizes are provided in Table 1.

Mercury isotope values (δ^202^Hg, Δ^199^Hg, Δ^200^Hg) differed among types of aquatic biota and habitats (river, delta, lake) in the study area (Table 1; Fig. 2). Fish from the Athabasca River and River Otter from the PAD had similarly low values of δ^202^Hg and Δ^199^Hg, with means ranging from − 0.85 to -0.78‰ and 0.57 to 0.68‰, respectively. More positive and variable values of δ^202^Hg (-0.55 to -0.12‰) and Δ^199^Hg (1.13–1.97‰) were observed in fish from western Lake Athabasca and Common Tern eggs from the delta and Lake Athabasca. Caspian Tern eggs from western Lake Athabasca showed the largest variation in MDF and odd MIF (Supplemental Fig. S1). The highest average values of δ^202^Hg (mean ± SE = 1.00 ± 0.10‰) and Δ^199^Hg (4.05 ± 0.14‰) were observed in Herring Gull eggs from eastern Lake Athabasca. Note the tern and gull species were all fish-eating birds. Overall, only small-magnitude variation of even MIF (Δ^200^Hg) was observed among biota, which is typical. Nonetheless, Herring Gull eggs from eastern Lake Athabasca had a significantly higher Δ^200^Hg (0.09 ± 0.01‰) compared with river preyfish (shiner species; 0.01 ± 0.01‰) and river Walleye (0.01 ± 0.01‰) (Tukey’s *p* ≤ 0.012, Supplemental Fig. S2, Table S1). Statistical results for pair-wise comparisons of mean Hg isotope values of biota are provided in Supplemental Table S1.

**Table 1:** Mercury concentrations and mercury stable isotope values of biota from the Athabasca River, Peace-Athabasca Delta, and Lake Athabasca. Values are means ± 1 standard deviation with the minimum and maximum in parentheses.

Biota	Matrix	Year	*N*	[THg] (µg/g dw)	δ^202^Hg (‰)	Δ^199^Hg (‰)	Δ^200^Hg (‰)
*Athabasca River*							
Preyfish^a^	Whole body	2018	11	0.19 ± 0.08 (0.08, 0.33)	−0.78 ± 0.11 (−96, -0.60)	0.67 ± 0.17 (0.47, 1.02)	0.01 ± 0.07 (−0.09, 0.09)
Walleye^b^	Muscle	2014	36	2.47 ± 1.11 (0.70, 5.22)	−0.80 ± 0.11 (−0.99, −0.53)	0.57 ± 0.21 (0.14, 0.98)	0.01 ± 0.05 (−0.12, 0.14)
*Peace-Athabasca Delta*							
River Otter^c^	Muscle	2014-15	6	2.02 ± 1.40 (0.71, 4.69)	−0.85 ± 0.17 (−1.02, −0.60)	0.68 ± 0.36 (0.05, 1.12)	0.02 ± 0.02 (−0.01, 0.05)
Common Tern^d^	Egg	2012-19	35	1.08 ± 0.28 (0.64, 1.72)	−0.28 ± 0.15 (−0.55, −0.04)	1.41 ± 0.31 (0.81, 2.35)	0.04 ± 0.05 (−0.08, 0.15)
*Lake Athabasca*							
Preyfish^a^	Whole body	2017-18	6	0.16 ± 0.06 (0.11, 0.27)	−0.37 ± 0.12 (−0.53, −0.18)	1.91 ± 0.17 (1.62, 2.11)	0.04 ± 0.03 (0, 0.08)
Walleye^b^	Muscle	2014	9	1.68 ± 1.34 (0.61, 4.76)	−0.55 ± 0.30 (−0.92, −0.04)	1.37 ± 1.18 (0.53, 4.09)	0.02 ± 0.08 (-0.13, 0.09)
Common Tern^d^	Egg	2011-22	99	1.47 ± 0.66 (0.46, 3.35)	−0.12 ± 0.37 (−1.66, 1.17)	1.97 ± 0.50 (1.07, 3.47)	0.04 ± 0.04 (−0.07, 0.16)
Caspian Tern^e^	Egg	2009-22	132	2.01 ± 0.87 (0.60, 5.21)	−0.33 ± 0.29 (−1.17, 1.07)	1.13 ± 0.45 (0.06, 2.91)	0.04 ± 0.04 (−0.06, 0.21)
Herring Gull^f^	Egg	2014	10	1.36 ± 0.46 (0.91, 2.43)	1.00 ± 0.32 (0.32, 1.42)	4.05 ± 0.47 (3.34, 4.99)	0.09 ± 0.02 (0.04, 0.11)

Latin names for sampled species are: ^a^ *Notropis* spp. ^b^ *Sander vitreus*, ^c^ *Lontra canadensis*, ^d^ *Sterna hirundo*, ^e^ *Hydroprogne caspia*, ^f^ *Larus argentatus*.

The large range of Δ^199^Hg (0.05–4.99‰) among biota suggested an environmental gradient in photochemical degradation of aqueous MeHg across habitats in the study area. The slope of odd MIF (Δ^201^Hg versus ^Δ199^Hg) was 1.28 ± 0.01 (r^2^ = 0.99, *p* < 0.001, *n* = 344) (Supplemental Fig. S3), which is consistent with laboratory observations of isotopic fractionation from photodemethylation of aqueous MeHg^46–48^. The mean (± SE) of biotic Δ^199^Hg in the Athabasca River (0.59 ± 0.08‰) was significantly lower (*p* < 0.001) than for biota in the delta (1.30 ± 0.09‰) and western Lake Athabasca (1.50 ± 0.04‰). Thus, bioaccumulation of less photodegraded MeHg occurred in the Athabasca River compared with downstream receiving waters. In this dataset, biotic δ^202^Hg was positively correlated with Δ^199^Hg, due to MDF during photochemical degradation of aqueous MeHg (Pearson *r* = 0.65, *p* < 0.001, *n* = 334). After correction of δ^202^Hg values for MDF during MeHg photodegradation (see Methods), we found River Otter, lake fish, and river fish did not differ significantly in δ^202^Hg_corr_ (means = -1.08 to -0.99‰), whereas eggs of terns species had higher δ^202^Hg_corr_ (-0.76 to -0.70‰) and Herring Gull eggs had the highest mean value (-0.27‰) (Supplemental Fig. S2, Table S1). A weak positive correlation was found between the Δ^199^Hg and Δ^200^Hg of biota (Pearson *r* = 0.35, *p* < 0.0001, *n* = 344), which suggested there was a tendency for greater source contributions of mercury from precipitation (positive Δ^200^Hg) in areas where greater photodegradation of MeHg occurred (positive Δ^199^Hg)^32^.

## River and lake water contributions to mercury accumulation in biota

Bayesian mixing model estimates derived from gradients in mercury isotopes indicated that downstream biota in the PAD and western Lake Athabasca bioaccumulated mercury that was primarily from the Athabasca River. Two water sources of bioavailable mercury were modelled to evaluate contributions to downstream aquatic food webs: (1) the Athabasca River, which discharges into the PAD and western Lake Athabasca, and (2) waters of eastern Lake Athabasca, which move westward and mix with inflows of the Athabasca River and PAD prior to draining into the Slave River (Fig. 1). Fish from the Athabasca River and Herring Gull eggs from eastern Lake Athabasca were used to estimate the Hg isotope signatures (Δ^199^Hg, Δ^200^Hg) of biologically-available river and lake mercury pools, respectively. As consumers, those biota provided integrated isotope signals of the mercury entering food webs in the river and lake waters. Bioaccumulated mercury from the Athabasca River had low Δ^199^Hg and near-zero Δ^200^Hg values, while bioaccumulated mercury from eastern Lake Athabasca had higher Δ^199^Hg and Δ^200^Hg (Supplemental Fig. S2, S4). The isotope mixing model estimated that the Athabasca River was the main source of bioaccumulated mercury in River Otter (mean [95% credible interval] = 94% [85–99%]) and Common Tern eggs (78% [73–82%]) collected from the PAD. In western Lake Athabasca, river contributions were also the main source of mercury for Walleye (85% [71–97%]), Caspian Tern eggs (84% [80–87%]), preyfish (64% [52–74%]), and Common Tern eggs (62% [58–66%]). Mixing model results were compared for different combinations of Hg isotope tracers (δ^202^Hg, δ^202^Hg_corr_, Δ^199^Hg, Δ^200^Hg), and all models showed similar results, with consistently dominant contributions of mercury from the Athabasca River (Supplemental Table S2).

**Fig. 3:**
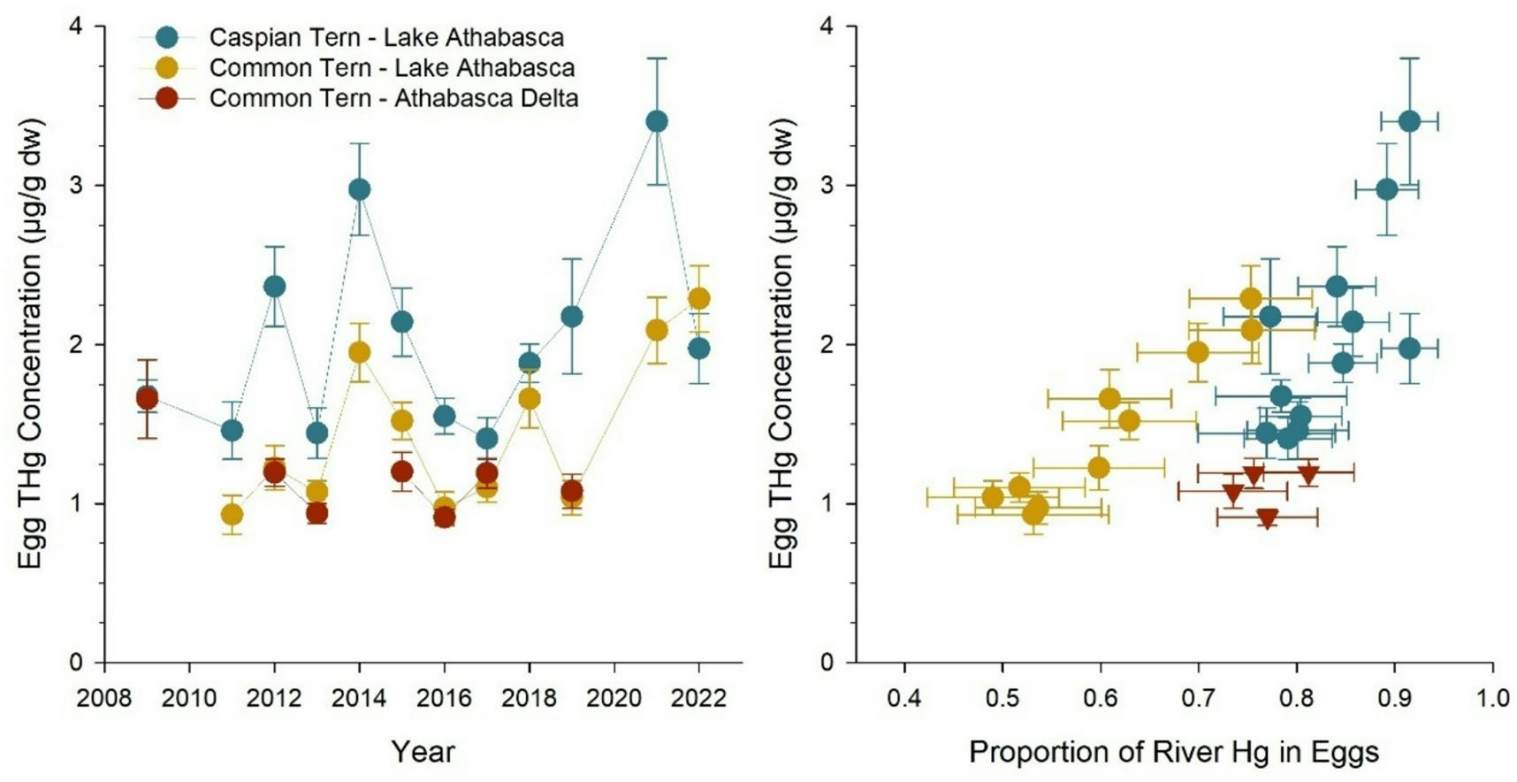
Inter-annual variability of THg concentration in eggs of Common and Caspian Terns, and relationships with the modelled contribution of mercury originating from the Athabasca River. Data are means of 10 eggs per year (sample sizes varied slightly in some years) and error bars are standard errors for THg concentration and 95% credible intervals for modelled river contributions.

There was large inter-annual variation of THg in eggs of terns breeding downstream in the PAD and western Lake Athabasca (Fig. 3). For each species and collection site, tern eggs varied approximately 2-fold in THg concentration during the period of 2009 to 2022, with higher mean concentrations in Caspian Tern eggs (2.01 ± 0.06 µg/g) than in Common Tern eggs (1.47 ± 0.7 µg/g) from Lake Athabasca and Common Tern eggs from the PAD (1.08 ± 0.13 µg/g) (one-way ANOVA, Tukey’s *p* < 0.001). Mixing model estimates indicated that annual mean concentrations of egg THg were positively correlated with the proportion of mercury originating from the Athabasca River for Caspian Tern (Pearson *r* = 0.73, *p* = 0.007) and Common Tern from Lake Athabasca (Pearson *r* = 0.96, *p* < 0.001) (Fig. 3). No correlation was observed for Common Tern eggs from the PAD, although the sample size was small (*n* = 4 years). Similarly, THg concentrations of Caspian and Common Tern eggs from Lake Athabasca were positively correlated with the flow (m^3^/s) of the Athabasca River prior to the sampling year (Supplemental Fig. S5) as well as the water level of Lake Athabasca (Supplemental Table S3). Together, the positive correlations of egg THg concentration with (1) the proportion of mercury originating from the river, and (2) inter-annual variability of river flow and lake water level, showed a dominant effect of mercury transport from the Athabasca River on bioaccumulation in downstream receiving waters.

Foraging and diet variation among individual birds also influenced the THg concentration of tern eggs. A linear model identified four significant explanatory variables of log THg in individual eggs: egg Δ^199^Hg, egg δ^13^C, egg δ^15^N, and Athabasca River flow (model r^2^ = 0.40, *p* < 0.001, *n* = 265; Supplemental Table S4). Other explanatory variables, specifically egg δ^202^Hg_corr_ and Lake Athabasca water level, were not significant (*p* > 0.05). While there was an overall trend of higher THg in eggs with lower Δ^199^Hg and in years after high river flow (Supplemental Fig. S5, S6), Δ^199^Hg ranged ~ 1.5-2‰ among eggs in any given year, which indicates variation in habitat foraging among individual birds in this large ecosystem. The diet of female birds at the time of breeding was important, as indicated by carbon and nitrogen stable isotope values of eggs. Eggs with higher δ^15^N had more THg due to biomagnification of mercury with increasing trophic position. Eggs with less negative δ^13^C also had more THg, indicating differences in dietary exposure to mercury in relation to carbon source. Together, these results showed individual birds foraged in a heterogenous lake environment, and feeding behaviour (foraging habitat, prey trophic position) affected their mercury exposure.

## River loads of mercury to downstream receiving waters

Long-term monitoring of surface water in the lower Athabasca River by the Government of Alberta showed large seasonal and inter-annual variation in mercury concentrations (Supplemental Datafile). Monthly-averaged concentrations in unfiltered river water ranged from 0.08 to 19.35 ng/L of THg (global mean = 3.33 ng/L, *n* = 548, from 2008 to 2022) and 0.02–0.40 ng/L of MeHg (global mean = 0.09 ng/L, *n* = 391, from 2013 to 2022). The highest concentrations were typically observed in May, June, and July (and to a lesser extent in August), coincident with the period of high discharge (Supplemental Fig. S7). Mean annual concentrations of THg (2.07–5.03 ng/L) and MeHg (0.06–0.12 ng/L) varied two-fold over a 15- and 10-year period, respectively. Annual loads of mercury from the Athabasca River to the PAD and Lake Athabasca were estimated at 111 ± 49 kg/year of THg (range = 39–210 kg/year) and 3.0 ± 1.3 kg/year of MeHg (range = 1.0–5.4 kg/year). On average, more than 80% of the annual mercury load was discharged during the high-water period from May to August (Supplemental Datafile). Inter-annual variation of THg in Caspian Tern eggs was positively correlated with the MeHg load of the Athabasca River from May to August in the previous year (Pearson *r* = 0.77, *p* = 0.027, *n* = 8). No correlation was observed between THg load from the river and THg concentration of Caspian Tern eggs, nor between THg or MeHg loads and THg in Common Tern eggs (*p* > 0.05).

**Fig. 4:**
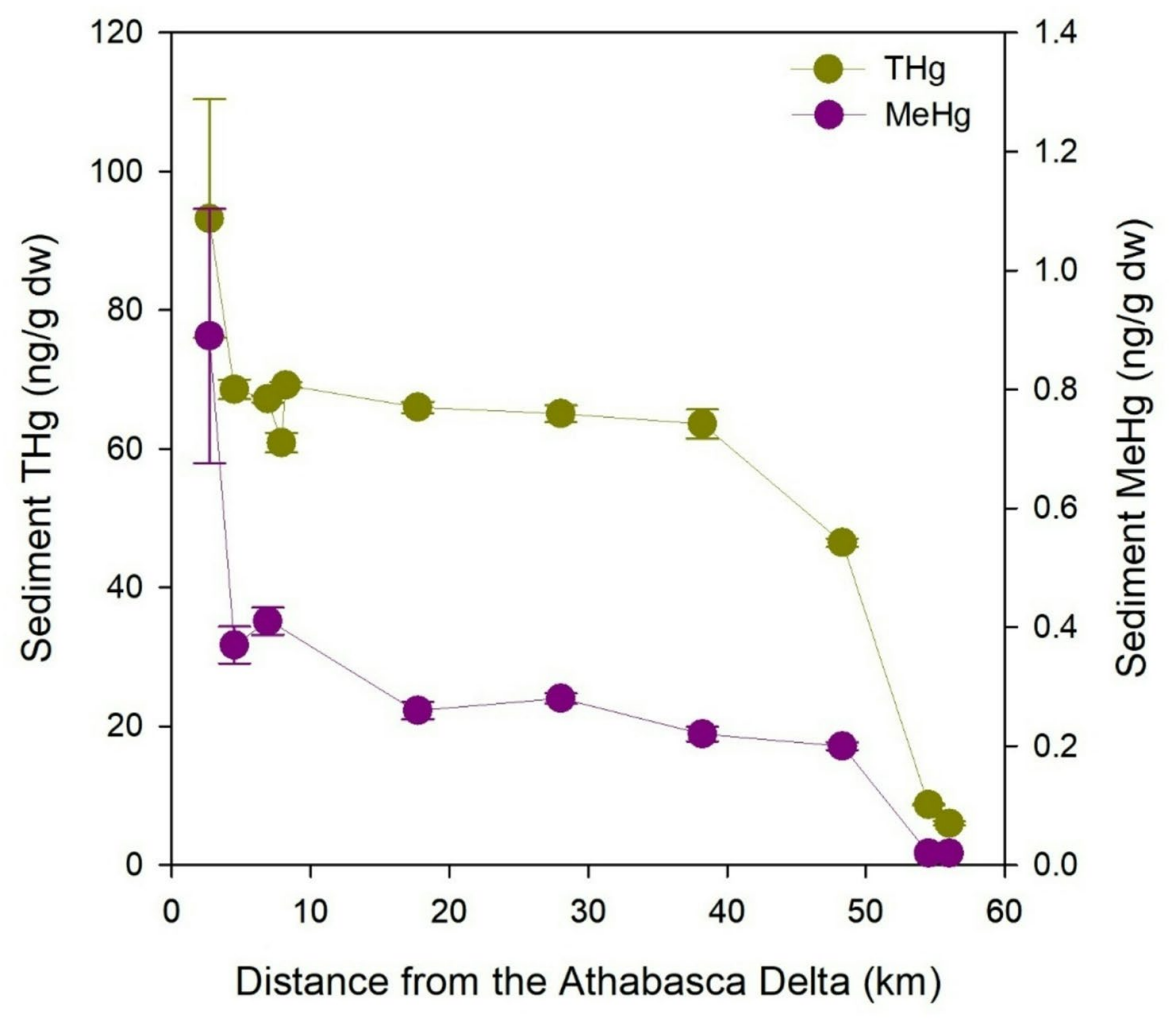
Gradients of THg and MeHg in surface sediment of western Lake Athabasca with increasing distance from the outflow of the Athabasca River. Data points are means ± standard error of triplicate samples.

A transect extending ~ 60 km from the Athabasca River mouth into western Lake Athabasca was sampled in July 2023 to examine the river-lake mixing zone. Along this transect, gradients were observed for mercury accumulation in sediments and plankton at the base of the food web. The THg and MeHg content of surface sediments was highest at sites < 10 km from the Athabasca Delta and decreased with distance from the inflow (Fig. 4). The lowest THg and MeHg concentrations were at the two farthest sites from the delta, where sediment MeHg concentrations were below analytical detection (< 0.04 ng/g). The total organic carbon (TOC) content of sediment also decreased with distance from the delta, and there was a strong positive correlation between sediment TOC and concentrations of both THg (Pearson *r* = 0.79, p 0.004, *n* = 11 sites) and MeHg (Pearson *r* = 0.96, *p* < 0.001, *n* = 9 sites). The median particle size of surface sediment varied among sites (7.0–86.4 μm, by volume), and there was a weak tendency of higher sediment THg concentration at sites with larger particles (Pearson *r* = -0.62, *p* = 0.041, *n* = 11 sites). The later trend reflected the rapid settling out of higher THg concentration (large) particles settling close to the delta. The mercury isotope values of Lake Athabasca surface sediment (δ^202^Hg, Δ^199^Hg) were not correlated with sediment characteristics (TOC, particle size) or distance from the delta. Food web uptake of MeHg was examined using water column samples of plankton (biomass > 200 μm). Higher plankton MeHg concentrations (mean ± SD = 24 ± 2 ng/g) were observed at sites < 10 km from the Athabasca Delta, and plankton concentrations were approximately 50% lower at sites farther away (11 ± 2 ng/g) (Supplemental Fig. S8). Together, these measurements of mercury in surface sediment and plankton showed evidence of river influence in western Lake Athabasca.

## Mercury isotope characterization of abiotic sources in the Athabasca River

**Fig. 5:**
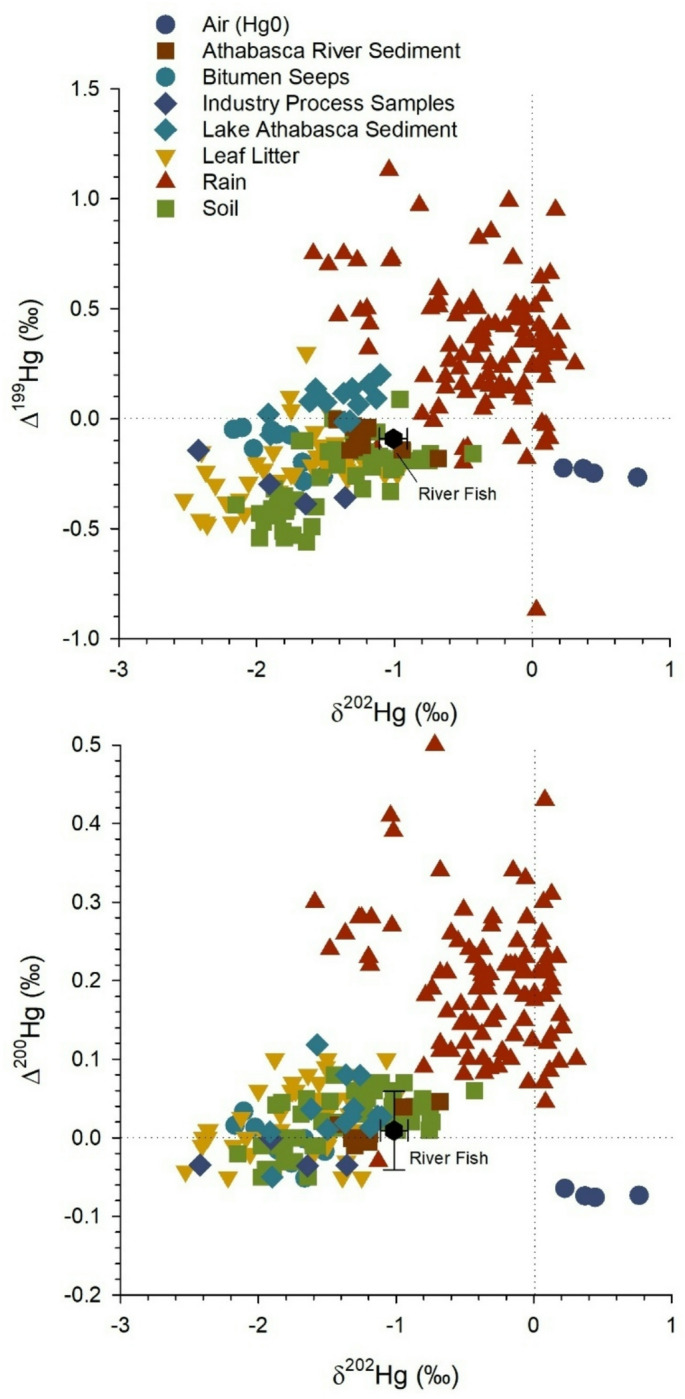
Isotope biplots (δ^202^Hg, Δ^199^Hg, Δ^200^Hg) of mercury in air, sediment and oil sands industry samples from the Athabasca River Basin. Data for leaf litter, rain, and soil are also included from the published literature for sites across North America (Supplemental Datafile contains data sources). Average δ^202^Hg_corr_ and Δ^200^Hg values of preyfish and Walleye from the Athabasca River are included for comparison with potential abiotic mercury sources. Mean fish δ^202^Hg_corr_ values (± 1 standard deviation) were corrected for MDF during photochemical processing (see Methods for details).

Large MDF and MIF of mercury isotopes were evident for potential abiotic sources of mercury in the Athabasca River Basin (Fig. 5). Mercury isotope data for air, river and lake sediment, bitumen seeps, and oil sands industry process samples were measured in the study area while data for leaf litter, rain, and soil were taken from the published literature for sites across North America (Supplemental Table S5, Datafile). The Hg isotope signatures of terrestrial samples were clustered together, namely sediment, soil, leaf litter and bitumen seeps, with negative values of δ^202^Hg and Δ^199^Hg. The similarity of mercury isotope values of river sediment with soil and leaf litter are consistent with erosional processes, whereas some lake surface sediments tended to have slightly more positive Δ^199^Hg values, possibly resulting from photochemical reduction of inorganic mercury. Bitumen seeps and industry process samples had similar isotope values to terrestrial matrices from across North America (i.e., leaf litter, soil) although they were more distinct from measured Athabasca Lake and Athabasca River sediment. Rain generally had positive Δ^199^Hg and higher δ^202^Hg values than terrestrial matrices. Air, as Hg(0), had positive δ^202^Hg and negative Δ^199^Hg values. The clustering of abiotic matrices described above was similar in Δ^200^Hg and δ^202^Hg isotopic space (Fig. 5).

The mercury isotope values of Walleye and preyfish from the Athabasca River were included for comparison with abiotic sources (Fig. 5). Note the mercury isotopes of fish were analyzed in tissue that contained primarily MeHg (> 85%, Supplemental Table S6) while abiotic matrices contained primarily or entirely inorganic mercury. Isotopic data for aqueous MeHg sources to the base of the food web were not measured. The one mercury isotope signature that showed clear separation between sources was Δ^200^Hg, which is not expected to undergo MIF once deposited on the landscape. The low values (~ 0.01‰) of fish Δ^200^Hg were consistent with predominately terrestrial sources of mercury (soil, sediment) and different from positive values of inorganic mercury in rain. The mean δ^202^Hg_corr_ of river fish (corrected for MDF during photochemical processes) was − 1.0 ± 0.1‰ and overlapped with δ^202^Hg values of river sediment, soils, leaf litter and rain. However, δ^202^Hg comparisons between biota and abiotic sources should be interpreted with caution because of MDF that can occur during biogeochemical cycling of mercury, including microbial methylation and demethylation processes in particular. Overall, the Δ^200^Hg measurements provided the strongest evidence that mercury in river fish was predominately of terrestrial origin.

## Discussion

### Mercury isotopes connect river transport with downstream bioaccumulation

Food webs in the PAD and western Lake Athabasca were exposed to mercury that originated predominately from the Athabasca River, which is the largest source of water entering both the delta and Lake Athabasca^39,49,50^. Several processes could explain the connection between the transport of river mercury and food web exposure downstream. From the river, large loads of dissolved and particulate inorganic mercury enter the delta and western Lake Athabasca each year, which may be transformed into MeHg by microbes in the water column and sediments^16^. The river supply of organic matter may also provide substrate that stimulates microbial activity and subsequent MeHg production in downstream environments^51,52^. A considerable load of aqueous MeHg from the Athabasca River enters the delta and western Lake Athabasca each year, where it may be taken up by microorganisms at the base of the food web. In Alberta rivers, including the Athabasca River, unfiltered water MeHg concentration is the primary correlate with fish mercury burdens^53^. The Athabasca River could also have an indirect effect on downstream bioaccumulation by flushing aqueous MeHg produced in shallow wetlands of the PAD to western Lake Athabasca^54,55^.

Several lines of evidence suggest river transport of MeHg was important for downstream bioaccumulation. All the biotic mercury isotope measurements were done on MeHg-rich tissues (i.e., muscle, egg, preyfish whole body have > 85% MeHg – Supplemental Table S6) and it has been experimentally demonstrated that MeHg does not undergo MDF and MIF during trophic transfer^19,24,56^, . Therefore, the biotic data represent the isotope signatures of MeHg pools entering the river, delta and lake food webs. The similarity of mean δ^202^Hg_corr_ values for preyfish and Walleye in the Athabasca River, River Otter in the PAD, and Walleye and preyfish in Lake Athabasca suggested a similar MeHg water source (the river) for all those biota. Tern eggs had more positive δ^202^Hg_corr_ values though still closer to river fish than to Herring Gull eggs from eastern Lake Athabasca. Thus, terns accumulated a mixture of MeHg from the river as well as other downstream MeHg sources. Terns forage widely and may travel distances from approximately 10–30 km to feed^57^, which may have contributed to mixed MeHg uptake. A second line of evidence – mixing model results based on two other isotopes (Δ^199^Hg and Δ^200^Hg signatures) – similarly indicated the Athabasca River was an important water source of MeHg. A third line of evidence was the positive correlation between MeHg load of the Athabasca River (but not THg load) and inter-year variation of THg in Caspian Tern eggs. Together, these results suggest the transport of aqueous MeHg from the Athabasca River contributed to food web exposure in downstream receiving waters. However, the river also delivers large amounts of inorganic mercury, which could be methylated in situ in the delta and lake. The wide variation of Δ^199^Hg, carbon and nitrogen isotope values among tern eggs also underscores the role of differential feeding within the mixing zone and nearshore versus offshore habitats for mercury exposure to individual birds. Future work involving the measurement of compound-specific isotope analysis of MeHg on abiotic and biotic matrices^58–60^ is recommended to more definitely track MeHg production and transport in the Athabasca River Basin.

## Sources of inorganic mercury and methylmercury in the Athabasca River Basin

The PAD and western Lake Athabasca are mixing zones that receive mercury from multiple sources including the Athabasca River, direct atmospheric deposition, and other hydrological inflows. Sediment-bound mercury from the Athabasca River and western Lake Athabasca had isotopic signatures consistent with mercury in terrestrial soil and organic matter reported for North America. Concentrations of suspended solids and particulate organic carbon are high in the Athabasca River (due to erosional inputs from tributaries and banks of the river mainstem) and vary seasonally with flow^61^. These terrestrial sources of mercury originate primarily from uptake of air Hg(0) in vegetation, which is the dominant flux to land surfaces^62–65^. While we cannot identify with certainty the source(s) of MeHg that bioaccumulated in river fish, their similar δ^202^Hg_corr_ and Δ^200^Hg values with abiotic matrices suggests that watershed production of MeHg in soils and wetlands, or in-river methylation of inorganic mercury from those sources, is important. Inorganic mercury bound to plant-derived organic matter can be methylated in boreal wetlands and shallow depressions^13^, then flushed into tributaries and the Athabasca River during high flows^43^. Runoff off from boreal forest has a similar mercury isotope signature to its soils^9^. Additionally, agricultural areas within the watershed may supply sediment-bound MeHg to the Athabasca River^53^, and riparian zones of large rivers are other sites of MeHg production^66,67^.

Natural bitumen seeps sampled within the Athabasca River Basin and oil sands industry process samples had mercury isotope signatures that fell within the cluster of terrestrial soils and leaf litter. Our new measurements of bitumen and industry samples were consistent with previous isotopic data generated a decade ago by Blum et al.^68^, which showed overburden, road material, processed sand, bitumen and mined oil sand had negative Δ^199^Hg values (~-0.6 to 0‰) and negative δ^202^Hg values (~-1.7 to -1.2‰). The similarity of mercury isotope signatures for terrestrial matrices and oil sands-related samples precluded an estimation of contributions from oil sands operations (including fugitive dust and soil erosion) to mercury bioaccumulation in river fish or biota downstream. Other lines of evidence provide additional information on this issue. Oil sands operations emit mercury that deposits on the landscape (typically within 30 km of sources), which may then flush into the Athabasca River via snowmelt and surface runoff^40,42,43^. Mercury emissions from oil sands processing facilities declined between 2007 and 2017^40^, though fugitive dust emissions and erosional inputs from operations are poorly constrained. The mercury content of bitumen and oil sands process samples, including petcoke, is relatively low (typically < 10 ng/g; Supplemental Table S5). Surface water monitoring over the last decade did not show changes in water THg and MeHg concentrations between reaches of the Athabasca River upstream and downstream of oil sands operations^61^. Evans and Talbot^69^ did not find evidence of enhanced mercury bioaccumulation in fish of the Athabasca River near oil sands operations. Estimates by Wasiuta et al.^43^ of mercury loads from tributaries impacted by oil sands mining were small in comparison with load estimates for the Athabasca River. Specifically, their recent estimates indicated 6 tributaries released a total of ~ 3 kg of THg per year and ~ 0.08 kg of MeHg per year, which accounts for < 10% of our THg and MeHg load estimates for the Athabasca River^43^. It is important to note that commercial mining of oil sands has occurred in the Athabasca River Basin since 1967 with steady increases in production after the year 2000^70^, and the above discussion only relates to recent environmental conditions. Further, the measurements reflected average conditions that may not have captured episodic short-term fluxes from anthropogenic activities or long-term accumulation of mercury in watershed compartments.

## Implications for understanding the fate of river transport in the mercury cycle

Mercury loads from the Athabasca River enhanced bioaccumulation in the eggs of terns breeding in western Lake Athabasca. As much as a doubling in egg THg concentration was observed between low and high flow years, with strong positive correlations connecting the relative contribution of river mercury and concentrations in eggs. The highest mean THg concentrations in Caspian Tern eggs (2.9–3.4 µg/g dw) were above toxicity thresholds associated with a risk of impairment to reproduction^71,72^. This finding of enhanced mercury bioaccumulation is based on many observations over a 14-year measurement period, which underscores the complexity of evaluating interactions between river transport and food web bioaccumulation. Considerable research has focused on river contributions to mercury biogeochemical cycling at regional and global scales^3,73–75^. Less information is available on the consequences of river mercury supply for bioaccumulation in downstream food webs.

A key finding is that the inter-annual variation in the river’s MeHg load, not its inorganic Hg load, directly correlated with enhanced downstream bioaccumulation. This indicates that the river’s supply of pre-formed, bioavailable MeHg was a dominant driver of food web exposure, underscoring the importance of catchment processes that generate and mobilize MeHg. The load of MeHg delivered from the Athabasca River to the delta and lake (on average 3 kg/year) was comparable to other large boreal rivers in Canada^75,76^, and therefore, other systems receiving inputs of MeHg from large boreal rivers should be examined for enhanced bioaccumulation [ 73,77]. Inorganic mercury and MeHg are supplied to fresh waters in several geochemical pools with varying reactivity^78–80^. Dissolved organic matter of terrestrial origin (e.g., humic and fulvic compounds) stabilizes MeHg during river transport by reducing UV penetration in water, and the chemical composition of the organic matter also influences the rate of MeHg photodegradation and its bioavailability^81–85^. In the Athabasca River, the MeHg in fish sampled over a long reach showed little photodegradation (low Δ^199^Hg values), which reflects the high turbidity of river water from suspended particulates and DOC^61^. Recent experimental research has demonstrated the MeHg load from terrestrial catchments is likely to have a disproportionately higher contribution to downstream bioaccumulation in estuaries relative to inorganic mercury pools^78,86^ and the extent of impact is related to organic matter quality^80,87,88^. Further research is recommended to characterize zones of inorganic mercury methylation and the complex interactions between organic matter chemical composition and aqueous MeHg^16,85,89^ that affect its fate within the Athabasca River Basin.

Another key finding is that Lake Athabasca is a large lake (with a surface area of 7,850 km^2^) that has a catchment-dominated mercury cycle in its western basin^16,90^. The mercury isotope mixing model results and the declining THg and MeHg concentrations in surface sediment with distance from the inflow suggested that loads from the Athabasca River played a larger role than other mercury pathways such as atmospheric deposition. Other research recently estimated more than 1500 km^2^ of western Lake Athabasca (i.e. 20% of the lake surface area) is likely influenced by the river plume^91^, where both inorganic mercury and MeHg originate primarily from the inflow. In contrast, Herring Gull eggs from eastern Lake Athabasca displayed mercury isotope characteristics typical of a lake-dominated mercury cycle^16,90^. Herring Gull eggs had the highest Δ^199^Hg and Δ^200^Hg values due to extensive photodemethylation of aqueous MeHg in a transparent water column and inputs from atmospheric deposition of Hg(II)^30,32,92^. Moreover, δ^202^Hg_corr_ signatures in Herring Gull eggs were significantly different from all other biota, suggesting a unique source of MeHg. In eastern Lake Athabasca, within-lake MeHg production and cycling likely dominated food web exposure as observed in the Great Lakes^32,93^. However, additional multi-year sampling of eggs and food web components (fish, plankton) would help to better constrain the mercury isotope signatures in eastern Lake Athabasca. Lake morphometry played a role in the contrasting mercury cycles between the western and eastern basins. The western basin is shallow (depths < 10 m) and prone to sediment resuspension from wind seiches^38^, while the eastern basin is deep (maximum depth = 124 m) with smaller catchment influence from river inflows^39^. Similarly, the influence of tributaries in the North American Great Lakes is restricted to nearshore areas where rapid sedimentation of river-transported mercury is a dominant removal process^1,2^.

In summary, this study generated clear evidence from mercury stable isotopes, spatial patterns, and temporal trends that river loads enhanced mercury bioaccumulation in downstream receiving environments of the Athabasca River Basin. The uptake of atmospheric Hg(0) by terrestrial vegetation, the production of MeHg in soils and wetlands within the basin, and subsequent mobilization of MeHg to the Athabasca River are likely important transport processes. In addition to delivery pathways, mercury exposure to downstream breeding birds was related to individual diet and foraging habits, and inter-year differences in river flow. The immense spatial scale of this study is noteworthy, which required years of measurements and diverse expertise to link transport processes to the biological fate of mercury in a large river-delta-lake complex. The study findings have clear implications for environmental management: catchment processes and disturbances that increase mercury loads in the Athabasca River will increase mercury concentrations of aquatic biota in downstream receiving waters.

### Methods

#### Study area

The lower reaches of the Athabasca River Basin were investigated, including the Athabasca River (approximately 500 km from the town of Athabasca to the mouth), the Peace-Athabasca Delta, and Lake Athabasca (Fig. 1). The study area is in the boreal plains ecozone of northern Alberta (latitude ~ 55–59 °N), dominated by typically flat terrain, coniferous forest, acidic and nutrient poor soils, and numerous wetlands. The Athabasca River drains a watershed of 159,000 km^2^ of largely coniferous and deciduous forest with some agricultural, urban and industrial development. The mean flow of the Athabasca River is lowest in winter (165 ± 45 m^3^/s, December to February, 2000–2023) and highest in summer (1141 ± 454 m^3^/s, June to August, 2000–2023), though there is large inter-year variability in flow. Upon reaching the Athabasca sector of the delta, the Athabasca River drains into Lake Athabasca via multiple channels with complex hydrological connections (including perched wetlands). Lake Athabasca receives the inflow of the river at the southwest end of the lake, close to the lake outflow that subsequently merges with the Peace River to form the Slave River. The Athabasca River is the main source of surface water to the Athabasca sector of the delta and western Lake Athabasca (where biotic samples were collected), while smaller rivers and tributaries drain into eastern Lake Athabasca within the province of Saskatchewan^39^. It is possible for water and sediment from the Peace River to drain into the delta and Lake Athabasca; however, this occurs only in very rare instances (i.e., not annually) when the river level is higher than the water level of the lake and delta. The Birch River also flows into the delta, though its discharge is low and has a minor hydrological influence compared to the Athabasca River^38^. The western section of Lake Athabasca is shallow (depths < 10 m), while the eastern basin is relatively deep (typically > 50 m, maximum depth 124 m) (Canadian Hydrographic Service, chart CHS6310). Within the study area, there is one city (Fort McMurray, with a population of ~ 80,000) and two small communities (Fort Mackay and Fort Chipewyan, with populations < 1000). At the time of study, oil sands mining had a terrestrial footprint of approximately 1000 km^2^ (Fig. 1)^94^.

### Sampling

Representative vertebrate species of local aquatic food webs were obtained from archived sample collections of the Oil Sands Monitoring (OSM) program (Fig. 1). A total of 45 Walleye (*Sander vitreus*) were collected from four sites on the Athabasca River and one site on Lake Athabasca in 2016 and processed to obtain dorsal muscle using standard methods^95^. River Otter (*Lontra canadensis*) carcasses were obtained from local trappers collecting animals in the Peace-Athabasca Delta and sampled for hind muscle (*n* = 6, 2014–2015). Emerald Shiner (*Notropis atherinoides*) and Spottail Shiner (*Notropis hudsonius*) were collected between 2017 and 2019 through community-based monitoring and OSM. A total of 17 preyfish samples, i.e. shiners (pools of whole-body fish), were obtained from 5 sites on the Athabasca River and two sites on western Lake Athabasca. Eggs of aquatic-feeding terns and gulls were collected in June over multiple years from breeding sites on Mamawi Lake (Common Tern, *Sterna hirundo*, *n* = 65, 2009–2019), Egg Island on western Lake Athabasca (Caspian Tern, *Hydroprogne caspia*, *n* = 132, 2009–2022; Common Tern, *n* = 110, 2011–2022), and an unnamed island in eastern Lake Athabasca (Herring Gull, *Larus argentatus*, *n* = 10, 2014). Opportunistic sampling of Herring Gull eggs provided an estimate of the mercury isotope signature of piscivorous birds in eastern Lake Athabasca where bird surveys did not find colonial breeding sites for terns in that part of the lake (unpublished observations). Herring Gull eggs are a well-established indicator of food web mercury bioaccumulation that has been used for decades in other regions such as the Great Lakes^96^. Animal use protocols and collections were reviewed and permitted by Environment and Climate Change Canada and the Government of Alberta.

Surface sediment and plankton were surveyed by boat along a ~ 60 km transect in western Lake Athabasca on July 24–27, 2023 to characterize the mixing zone of river and lake waters. A total of 11 sites were sampled and the mouth of the Embarras River was used as the point of reference to estimate distance from the Athabasca Delta (river inflow to the lake). The two farthest sites were located outside the sediment plume (at the time of sampling) as indicated by high water clarity. At each site, surface sediments were sampled in triplicate with three separate Ekman grabs. Approximately 50–100 g wet weight of sediment was scooped per sample from the top 3 cm of the grab and placed in a whirlpak bag. Single or duplicate plankton samples were collected from each site by horizontal tows of a large plankton net (mesh size 200 μm, 1 m diameter opening) near the water surface. Plankton samples were placed in trace-metal clean 250 mL HDPE jars. Visual inspection of net contents suggested samples were composed primarily of zooplankton and macro-filamentous phytoplankton. Plankton were not collected from two shallow sites (~ 2 m depth) near the delta due to extensive macrophyte debris found in the net and water column. Sediment and plankton samples were frozen on the day of collection.

Abiotic samples of potential mercury sources in the Athabasca River Basin were collected for mercury isotope analysis. Passive sampling of atmospheric gaseous elemental mercury (GEM) was performed using MerPAS samplers^97^ obtained from Tekran Instruments Corporation (Toronto, Canada). Passive samplers were deployed at two locations, Fort McKay (nearfield site) and Stony Mountain Wildlife Nature Preserve (reference site), for approximately 3-month intervals from June to September 2022 and September to January 2023. Each deployment involved separate MerPAS samplers for measurement of THg concentration, mercury isotopes in air and a blank, all of which were attached to fencing at least 1 m from the ground. Samplers were sealed and double-bagged for transport and storage. Athabasca River sediment was obtained from archived collections of the Government of Alberta for 10 locations sampled along the length of the river from 1989 to 1992. Additionally, glacial till (*n* = 2) was obtained from the headwaters of the Athabasca River^98^. Natural bitumen seeps were sampled in June 2021 at three distinct locations near Fort McMurray (Hangingstone River, Gateway Hill, Crane Lake), where three soil samples were obtained at each site. Industry samples (*n* = 4) were obtained in 2016 with the cooperation of the Canadian Oil Sands Innovation Alliance and included unprocessed and processed bitumen, and petcoke.

### Laboratory analysis

Sediment, plankton, preyfish, muscle of Walleye and River Otter, and bird eggs were homogenized and freeze-dried prior to chemical analysis. Dried sediment, plankton and muscle samples were homogenized by acid-washed mortar and pestle, while preyfish and egg contents (shell excluded) were homogenized by ball-milling prior to drying. Sediment particle size was measured on separate samples of wet sediment with a Malvern Panalytical analyzer at SGS Environmental Services (Lakehead, Canada).

The total mercury (THg) concentration of samples was measured on a Direct Mercury Analyzer at the National Wildlife Research Centre, a facility of Environment and Climate Change Canada (Ottawa, Canada). Dried and homogenized aliquots of sediment (*n* = 33), plankton (*n* = 14), gull and tern egg (*n* = 317), preyfish (*n* = 17) and muscle of River Otter (*n* = 6) were combusted in a nickel boat, followed by gold amalgam trapping and detection by atomic absorption spectrometry. Note that most of the THg in eggs, preyfish and muscle is in the form of MeHg (Supplemental Table S6), and THg data for tern eggs collected from 2009 to 2017 were reported earlier^33^. For Walleye, THg was determined on wet homogenized muscle using the same methods (Direct Mercury Analyzer) at the National Hydrology Research Centre, a facility of Environment and Climate Change Canada (Saskatoon, Canada). All results were above the method reporting limit of 0.1 ng. Analytical duplicates and certified reference materials were analyzed every ten samples, and results deviated < 15% from expected values.

Methylmercury was measured at Flett Research Ltd (Winnipeg, Canada) in samples of sediment (*n* = 27), plankton (*n* = 14), preyfish (*n* = 26) and Walleye muscle (*n* = 20). Dried sample (20 mg) was digested in a potassium hydroxide-methanol solution followed by ethylation, purge and trap steps, separation by gas chromatography, and fluorescence detection according to United States Environmental Protection Agency (US EPA) method 1630. The method detection limit was 0.4 ng/g and concentrations were expressed on a dry weight basis. Recoveries of MeHg from a certified reference material (DORM-4, National Research Council of Canada) average 90 ± 7% (*n* = 6), and analytical duplicates had a relative percent difference of 7 ± 6% (*n* = 12).

Mercury isotope measurements on a subset of biological samples (*n* = 344) and on sediment from Lake Athabasca (*n* = 13) were performed by the Water Quality Centre at Trent University (Peterborough, Canada). The samples were digested using 3 mL of aqua regia acid in a 40 mL amber glass vessel by heating on a hot plate at 120 °C for 4 h. After digestion, the supernatant was extracted and made up to 20 mL with 0.002 M BrCl. Samples were diluted as needed to a final concentration of 5 ppb Hg. An Elemental Scientific Inc. (ES) ICP Hydride generation system was used to generate a Hg(0) vapor by the quantitative reduction of Hg(II) in solution using SnCl_2_ (3% w/v in 1 M HCl). The Hg(0) vapor was introduced directly into the mass spectrometer, with sample bracketing of the National Institute of Standards and Technology (NIST) SRM 3133 mercury standard, and a thallium internal standard was introduced for mass bias correction. The isotope measurement was performed on a Nu Plasma II multicollector inductively coupled plasma mass spectrometer (MC-ICP-MS).

Stable isotopes of atmospheric Hg(0) sorbed in passive air samplers were measured in the Department of Earth Sciences at the University of Toronto (Toronto, Canada). Method details are provided in Szponar et al.^97^. Briefly, Hg(0) sorbed to sulfur-impregnated activated carbon in MerPAS samplers (*n* = 4; see Sampling section above) was combusted and concentrated in an oxidizing trap solution containing 10% H_2_SO_4_ (v/v) and 0.21% potassium permanganate (KMnO_4_, w/w), then reduced with 30% (w/w) hydroxylamine hydrochloride and preserved with 2% bromium chloride. Samples were later analyzed for mercury isotopes with a MC-ICP-MS (Neptune Plus, Thermo Scientific). Following inline SnCl_2_ reduction, Hg(0) was introduced to the mass spectrometer with a thallium internal standard and sample bracketing of NIST SRM 3133 to correct for instrument mass bias.

Abiotic samples, specifically Athabasca River sediment (*n* = 12), natural bitumen seeps (*n* = 9) and industry samples (*n* = 4) were analyzed for mercury isotopes in the Department of Earth Sciences plasma mass spectrometry laboratory at the University of New Hampshire (Durham, United States of America) as per Demers et al.^64^. Mercury was extracted from samples by thermal reduction using a dual Lindberg Blue tube furnace assembly. Extracted mercury was trapped in 24 g solutions of 1% KMnO_4_ (w/w) in 10% H_2_SO_4_ (v/v). Secondary matrix separation and pre-concentration for isotopic analysis was done by semi-automated purge and trap into 1% KMnO_4_/10% H_2_SO_4_ trapping solutions with the Nippon MA2000. Trapping solutions were measured for Hg concentration using a Nippon RA3F cold vapor atomic fluorescence spectrometer in accordance with USEPA Method 1631 to match sample/standard run solution concentrations to within 5% prior to isotopic analysis. Combustion and secondary matrix separation performance was monitored with procedural blanks and reference materials. Procedural blanks typically contained < 1% of the mercury mass measured in samples, with a maximum contribution of 1.5%. We prepared NIST SRM 1632c (Bituminous Coal, 93.8 +/- 3.7 ng/g), achieving a combustion recovery of 103.2 +/- 0.3% (1SD, *n* = 2) and secondary matrix separation recovery of 99.2 +/- 1.8% (1SD, *n* = 2); we prepared NIST SRM 1944 (NY/NJ Waterway Sediment, 3,400 +/- 500 ng/g), achieving a combustion recovery of 104.2% (*n* = 1) and secondary matrix separation recovery of 99.4% (*n* = 1). All secondary matrix separations for sediment, bitumen, and industry samples recovered at 99.1 +/- 1.4% (1SD, *n* = 25). Mercury isotopic composition was measured using a MC-ICP-MS (Nu Instruments, Nu Plasma II) using continuous flow cold vapor generation with Sn(II) reduction^99,100^. Simultaneous measurements were made for masses 195, 196, 198–205, and 207 Instrumental bias was corrected using an internal Thallium standard (NIST SRM 997, ^205^Tl/^203^Tl ratio of 2.38714) introduced with an Aridus II desolvating nebulizer and strict sample-standard bracketing using NIST SRM 3133 Hg standard. On-peak zero corrections were applied to all masses.

All mercury isotope data generated from the three laboratories are reported as the per mil deviation from the xxxHg/198Hg ratio of the NIST SRM 3133 mercury standard using delta notation (δ) in units of per mil (‰) where xxx is the mass of the Hg isotope (199, 200, 201, 202, and 204):

δxxxHg (‰) = ([(xxxHg∕198Hg)_Sample_ ∕ (xxxHg∕198Hg)_NIST3133_]–1) × 1,000.

The magnitude of odd and even mass independent fraction was determined from the measured δ value and the kinetic mass dependent fractionation law using kappa notation (Δ) in units of per mil (‰):

ΔxxxHg = δxxxHg – (βxxx × δ202Hg).

where xxx is the mass of the Hg isotope (199, 200, 201, 202, and 204) and βxxx is the mass dependent scaling factor of 0.252 for ^199^Hg, 0.502 for ^200^Hg, 0.752 for ^201^Hg, and 1.493 for ^204^Hg as reported in Blum et al.^23^. Raw data, uncertainties, and reproducibility of standards for the mercury isotope measurements from all laboratories are provided in the Supplemental Datafile.

Carbon and nitrogen stable isotopes were measured on egg samples (*n* = 276) in the Jan Veizer Stable Isotope Laboratory at the University of Ottawa (Ottawa, Canada). Samples and standards were weighed in tin capsules and combusted in a Vario EL Cube elemental analyzer interfaced to a Thermo Delta Advantage isotope ratio mass spectrometer. The δ^13^C and δ^15^N results were reported as the per mil (‰) deviation from the Vienna PeeDee Belemnite and AIR standards, respectively. A duplicate and an internal standard were analyzed every 10 samples and QA/QC results were within 0.2‰.

### Data analysis

Biotic δ^202^Hg values were corrected for photochemical effects using a statistical approach modified from Janssen et al.^25^. First, the “lm” function in R program (R Core Team; v4.4) was used to generate a global model of δ^202^Hg as a function of Δ^199^Hg (continuous covariate) and biota (categorical variable, each species by habitat):

δ^202^Hg = 0.3075 (± 0.0304) * Δ^199^Hg + biota – 0.6760 (± 0.0409).

The model and coefficients were highly significant (model adjusted R-squared = 0.64, F-statistic = 69.74 on 9 and 334 DF, *p* < 0.0001). Individual biotic δ^202^Hg values were then corrected with the following equation assuming the modelled Δ^199^Hg-δ^202^Hg slope and a baseline Δ^199^Hg from Athabasca River sediment:

δ^202^Hg_corr_ = δ^202^Hg_biota_ – (m* (Δ^199^Hg_biota_ – Δ^199^Hg_sed_)).

where δ^202^Hg_biota_ and Δ^199^Hg_biota_ are the isotope values for a biotic sample, m = slope of Δ^199^Hg-δ^202^Hg (0.3075), and Δ^199^Hg_sed_ is the mean value for Athabasca River sediment (-0.08 ± 0.05‰, *n* = 10, Supplemental Table S5).

Surface water concentrations of THg and MeHg in the Athabasca River were obtained from the Water Quality Data Portal of the Government of Alberta (https://environment.extranet.gov.ab.ca/apps/WaterQuality/dataportal/). Data were available for six monitoring stations covering more than 500 km of the Athabasca River between Klondyke Ferry (Vega) and the Devils Elbow (near the delta). Three stations were in reaches of the river upstream of oil sands mines, and three stations were located downstream of the mines. Government sampling and analytical methods can be found in the Government of Alberta Water Quality Data Portal. Water concentrations of THg in the Athabasca River were available for the study period (2008–2022) while MeHg concentrations were available for a more restricted period (2013–2022). The number of measurements varied between stations and years, and a summary of the water concentration data is provided in the Supplemental Datafile. Seasonal, spatial and inter-annual variation of river water THg and MeHg concentrations were examined with data plots. We did not find spatial patterns of water mercury concentrations between the 6 stations, similarly noted by Glozier et al.^61^, and therefore data from all stations were pooled to generated monthly estimates of THg and MeHg concentrations in Athabasca River water over the study period. River discharge data were available for the study period from the Water Survey of Canada at one station (Station No: 07DA001, Station Name: Athabasca River below Fort McMurray) (https://wateroffice.ec.gc.ca/). Water inflow of six tributaries downstream of the discharge monitoring station were not represented in the estimates, though the tributary discharges were relatively small compared to the Athabasca River^43^. The mean monthly discharge (m^3^/s) of the Athabasca River and the mean monthly concentration (ng/L) were used to calculate loads of THg and MeHg in kg/month, accounting for the volume of water discharged over the entire month. The annual river load was then calculated as the sum of monthly loads for a given year. Detailed breakdown of the THg and MeHg load estimates of the Athabasca River by month and year are provided in the Supplemental Datafile.

Published mercury isotope data for a broader suite of abiotic matrices were obtained from the Global Observation System for Mercury Isotopes (iGOS4M). The publicly accessible iGOS4M Hg isotope database was accessed online (at https://sites.google.com/view/igos4m/database in November 2024) to obtain mercury isotope data for soil, leaf litter and rain compiled from published studies in Canada and the United States of America. Data details including original article sources are provided in Supplemental Datafile. Four anomalous Δ^200^Hg values (0.59, 0.74, 1.18, 23 per mil) for precipitation were excluded from the comparison.

Pearson correlations were examined in SigmaPlot (version 15, Grafiti LLC, Palo Alto, California, USA) to test for associations between mercury isotopes, biotic THg concentrations and environmental variables. The effect of hydrology (Athabasca River flow [m^3^/s], Lake Athabasca water level [m]) on inter-annual variability in tern egg THg concentrations were examined with Pearson correlations. Several time-dependent variations on those variables (i.e. mean annual, April-May of sampling year, June or May-August of year prior to sampling) were tested to account for possible seasonality of effects. Due to the large number of comparisons with hydrology variables, probability values were adjusted for the experiment-wide error rate using Holm’s adjustment. Linear models were tested in R program using the built-in “lm” function. Differences in mercury isotope values between habitat types (river, delta, lake) and between biota in those habitats were examined with linear models. Estimated marginal means and the significance of pair-wise differences (Tukey’s method) were determined with the “emmeans” package. Environmental drivers of THg concentrations in individual Tern eggs were examined with a linear model that included effects of diet (δ^13^C, δ^15^N), mercury source (Δ^199^Hg, δ^202^Hg), _corr_and hydrology (Athabasca River flow, Athabasca Lake water level).

Using MixSIAR in R^101^, a Bayesian mixing model was run to estimate the contributions of mercury pools from the Athabasca River versus Lake Athabasca to bioaccumulation, specifically for Caspian Terns (*n* = 132), Common Terns (*n* = 99), Walleye (*n* = 9) and preyfish (*n* = 6) from Lake Athabasca, and River Otter (*n* = 6) and Common Terns (*n* = 35) from the Peace-Athabasca Delta. Various combinations of four Hg isotope tracers (δ^202^Hg, δ^202^Hg_corr_, Δ^199^Hg, Δ^200^Hg) were tested in mixing models, and little difference was found in estimates of river and lake contributions to biota (see comparisons in Supplemental Table S2). Final mixing models were based on Δ^199^Hg and Δ^200^Hg, which do not under-go tissue-specific fractionation following biological incorporation. Signatures of Δ^199^Hg and Δ^200^Hg in the river endmember were obtained from measurements of preyfish and Walleye in the Athabasca River (mean Δ^199^Hg = 0.59 ± 0.21‰, mean Δ^200^Hg = 0.01 ± 0.06‰, *n* = 47) and for the lake endmember, mercury isotope signatures were obtained from Herring Gull eggs collected in eastern Lake Athabasca (mean Δ^199^Hg = 4.05 ± 0.47‰, mean Δ^200^Hg = 0.09 ± 0.02‰, *n* = 10). For both Δ^199^Hg and Δ^200^Hg, values in consumers were assumed to directly reflect the isotopic content of their diet in those habitats, with no fractionation associated with trophic transfer^19,102^. As such, observed variation in consumer Δ^199^Hg and Δ^200^Hg values can be attributed to shifts in the relative contributions of river and lake pools of mercury. Both models were run using the “very long” Monte-Carlo Markov Chain preset provided by MixSIAR (chain length = 1,000,000; burn-in = 500,000; thin = 300; no. chains = 3). Due to constraints within the MixSIAR framework, which allows for only two effects within a model, species and location were combined into a single fixed effect (“group”), while time was treated as random effect. Convergence of both models was assessed using Gelman-Rubin and Geweke diagnostic tests; all Gelman-Rubin values were < 1.01, and ~ 5% of Geweke test values fell outside ± 1.96 in each chain.

## Supplementary Information

Below is the link to the electronic supplementary material.

Supplementary Material 1

Supplementary Material 2

## Data Availability

Mercury stable isotope data used in this publication are available in the Supplementary Information.

## Email

john.chetelat@ec.gc.ca.

## Supplementary Information

This datafile contains mercury stable isotope measurements of abiotic and biotic matrices.
