# Supplementary material for: The Athabasca River regulates methylmercury burdens of waterbirds breeding downstream
Source: Sci Rep. 2026 Jan 17;16:5630. doi: 10.1038/s41598-026-35970-z (PMC12891722; doi:10.1038/s41598-026-35970-z)
Supplement: Supplementary file 2 — Supplementary Material 2 [file 41598_2026_35970_MOESM2_ESM.docx]

Supplementary Information for:

The Athabasca River regulates methylmercury burdens of waterbirds breeding downstream

John Chételat^1^*, Craig Hebert^1^, Jason Demers^2^, Colin A. Cooke^3,4^, Christine McClelland^1^, Maureen Angell^1^, Bridgit Bergquist^5^, Marlene Evans^6^, Maria F. Fahnestock^7^, Kuzey Güneşli^1^, Sarah Greenwood^1^, Bruce Maclean^8^, Mark McMaster^9^, Lukas Mundy^1^, Gerald Tetreault^9^, Philippe J. Thomas^1^

Affiliations

^1^ Environment and Climate Change Canada, National Wildlife Research Centre, Ottawa, Ontario, Canada

^2^ Earth Systems Research Center, University of New Hampshire, Durham, New Hampshire, USA

^3^ Environment and Protected Areas, Government of Alberta, Edmonton, Alberta, Canada

^4^ Earth and Atmospheric Sciences, University of Alberta, Edmonton, Alberta, Canada

^5^ Department of Earth Sciences, University of Toronto, Toronto, Ontario, Canada

^6^ Environment and Climate Change Canada, National Hydrology Research Centre, Saskatoon, Saskatchewan, Canada

^7^ Joan and James Leitzel Center for Mathematics, Science, and Engineering Education, University of New Hampshire, Durham, New Hampshire, USA

^8^ Maclean Environmental Consulting, Winnipeg, Manitoba, Canada

^9^ Environment and Climate Change Canada, Canada Centre for Inland Waters, Burlington, Ontario, Canada

*John Chételat

**Email:**  [john.chetelat@ec.gc.ca](mailto:john.chetelat@ec.gc.ca)

Contents

[**Figure S1.** Mercury isotope values of aquatic biota from the Athabasca River, Peace-Athabasca Delta and Lake Athabasca. 3](#_Toc205468308)

[**Figure S2.** Mean (± standard error) of mercury isotopes for aquatic biota organized by habitat type (river, delta and lake). 4](#_Toc205468309)

[**Table S1.** Tukey probability values for pairwise comparisons of mercury isotopes (δ^202^Hg_corr_, Δ^199^Hg, Δ^200^Hg) of aquatic biota, categorized by habitat type (river, delta and lake). 5](#_Toc205468310)

[**Figure S3.** Biplot of odd mercury isotopes (Δ^201^Hg, Δ^199^Hg) to assess photochemical degradation of methylmercury in biota from Lake Athabasca, the Peace-Athabasca Delta and the Athabasca River. 8](#_Toc205468311)

[**Table S2.** Comparison of Bayesian mixing model results for estimates of river and lake contributions to mercury in biota using different combinations of Hg isotopes (δ^202^Hg, δ^202^Hg_corr_, Δ^199^Hg, Δ^200^Hg) as source tracers. 9](#_Toc205468312)

[**Figure S4.** Endmember biplot (Δ^199^Hg, Δ^200^Hg) for the Bayesian mixing model of river and lake contributions of mercury to biota in Lake Athabasca (LA) and the Peace-Athabasca Delta (PAD). 11](#_Toc205468313)

[**Table S3.** Pearson coefficients for correlations between flow (m^3^/sec) of the Athabasca River or Lake Athabasca water level (m) and THg concentration (µg/g dw) of eggs of Caspian Tern and Common Tern from Lake Athabasca. 12](#_Toc205468314)

[**Figure S5.** Positive correlations between egg THg concentration and discharge of the Athabasca River (in June of the previous year) for Caspian Tern (Pearson r = 0.90, p < 0.001, n = 12) and Common Tern (Pearson r = 0.74, p = 0.009, n = 11) from Lake Athabasca. 13](#_Toc205468315)

[**Figure S6.** Inter-annual variation in egg THg concentration was negatively correlated with egg mercury isotope composition (Δ199Hg) for both Caspian Tern (Pearson r = -0.68, p = 0.015, n = 12) and Common Tern (Pearson r = -0.93, p < 0.001, n = 10) on Lake Athabasca. 14](#_Toc205468316)

[**Table S4.** Results of a linear model relating log-transformed THg concentration of Tern eggs to diet (δ^13^C [‰], δ^15^N [‰]), mercury source (δ^202^Hg_corr_ [‰], Δ^199^Hg [‰]) and hydrological variables (Athabasca River flow [m^3^/s], Athabasca Lake water level [m]). 15](#_Toc205468317)

[**Figure S7.** Mean surface water concentrations of total mercury, dissolved mercury and methylmercury in the Athabasca River at two sampling locations. 16](#_Toc205468318)

[**Figure S8.** Longitudinal variation of methylmercury concentration in plankton of Lake Athabasca with distance from the Athabasca Delta. 17](#_Toc205468319)

[**Table S5.** Mercury concentrations and mercury stable isotope values of abiotic matrices from the Athabasca Oil Sands Region. 18](#_Toc205468320)

[**Table S6.** Percent methylmercury (MeHg) content of biotic tissues examined in this study. 19](#_Toc205468321)

**Figure S1.** Mercury isotope values of aquatic biota from the Athabasca River, Peace-Athabasca Delta and Lake Athabasca. Symbols represent measurements on individual samples (n = 343).


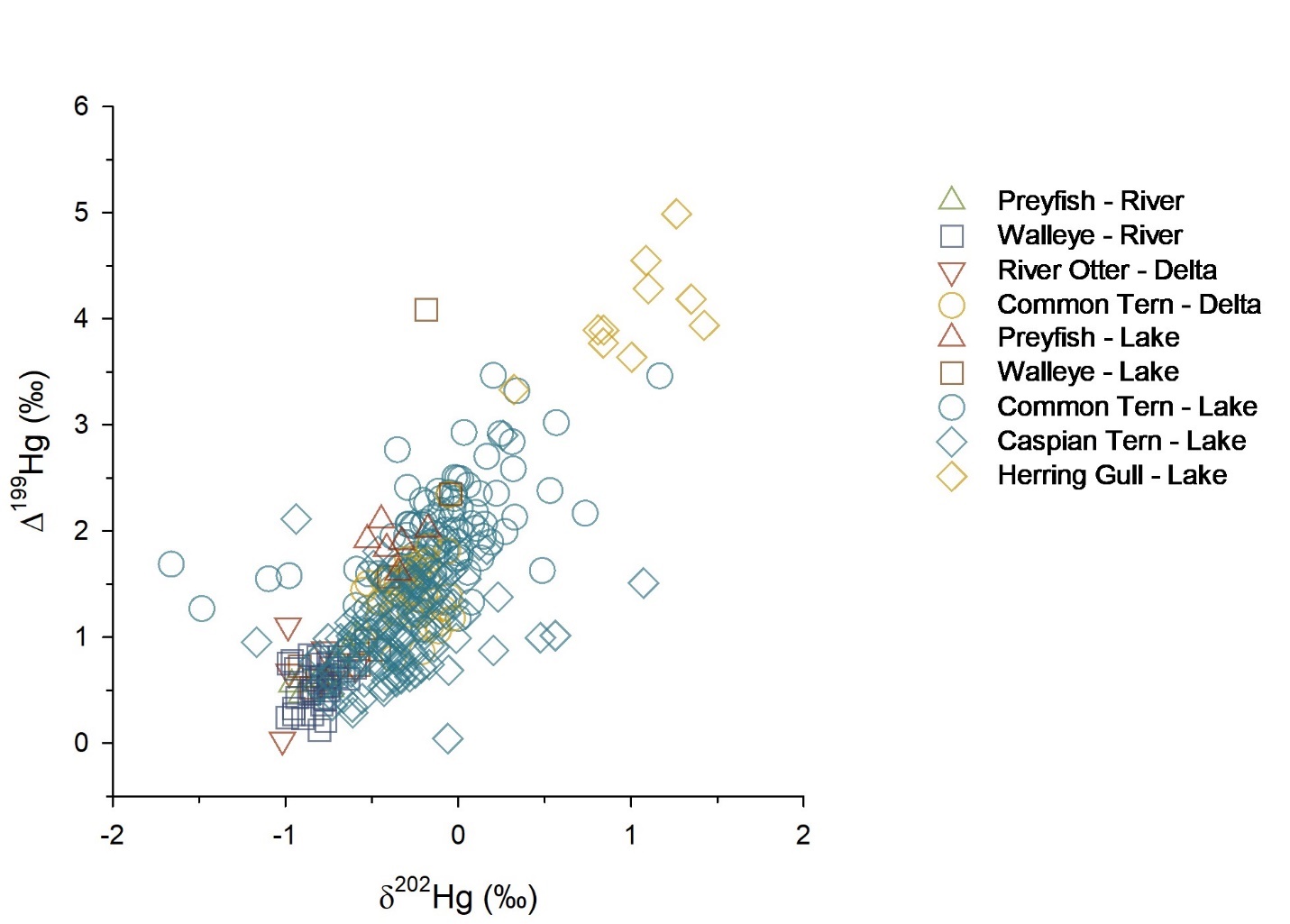


**Figure S2.** Mean (± standard error) of mercury isotopes for aquatic biota organized by habitat type (river, delta and lake). Statistical comparisons were performed with linear models, and pairwise comparisons were adjusted for the experiment-wise error rate using the Tukey method. See Table S1 for probability values of pair-wise comparisons. Mass-dependent fractionation of δ^202^Hg was corrected for effects of methylmercury photodegradation (see Methods for details). Samples sizes are provided in Table 1.


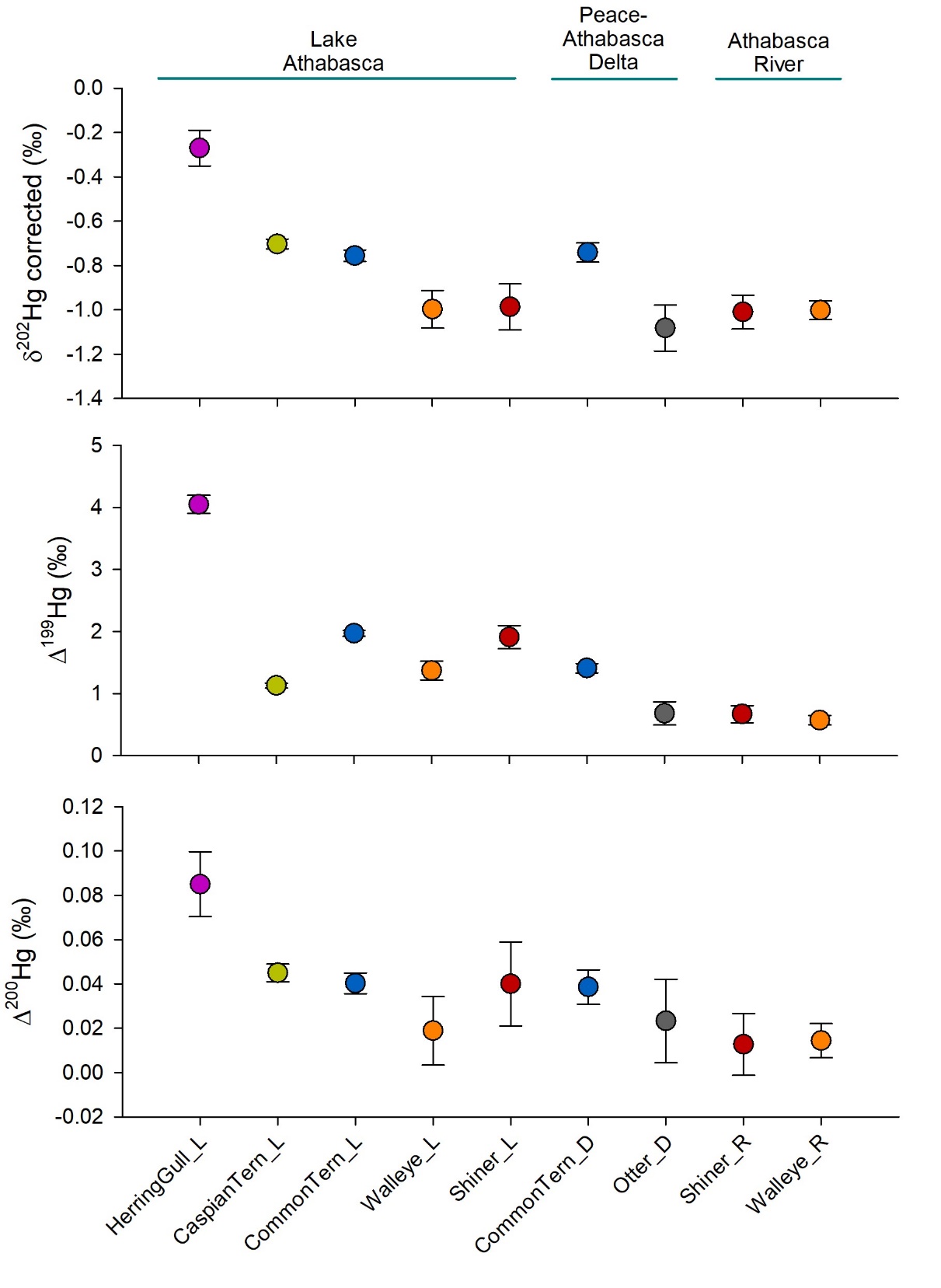


**Table S1.** Tukey probability values for pairwise comparisons of mercury isotopes (δ^202^Hg_corr_, Δ^199^Hg, Δ^200^Hg) of aquatic biota, categorized by habitat type (river, delta and lake). See Figure S2 for means and standard errors.

| **δ^202^Hg_corr_** |  |  |  |  |  |
| --- | --- | --- | --- | --- | --- |
| **contrast** | **estimate** | **SE** | **df** | **t.ratio** | **p.value** |
| CaspianTern_Lake-CommonTern_Lake | 0.05301 | 0.0338 | 335 | 1.569 | 0.8209 |
| CaspianTern_Lake-CommonTern_PAD | 0.03878 | 0.0483 | 335 | 0.803 | 0.9967 |
| CaspianTern_Lake-HerringGull_Lake | -0.43265 | 0.0833 | 335 | -5.192 | **<.0001** |
| CaspianTern_Lake-Otter_PAD | 0.37902 | 0.1061 | 335 | 3.574 | **0.0119** |
| CaspianTern_Lake-Shiner_Lake | 0.28402 | 0.1061 | 335 | 2.678 | 0.1597 |
| CaspianTern_Lake-Shiner_River | 0.30735 | 0.0797 | 335 | 3.855 | **0.0044** |
| CaspianTern_Lake-Walleye_Lake | 0.29402 | 0.0875 | 335 | 3.359 | **0.0243** |
| CaspianTern_Lake-Walleye_River | 0.29902 | 0.0478 | 335 | 6.259 | **<.0001** |
| CommonTern_Lake-CommonTern_PAD | -0.01423 | 0.05 | 335 | -0.285 | 1 |
| CommonTern_Lake-HerringGull_Lake | -0.48566 | 0.0843 | 335 | -5.761 | **<.0001** |
| CommonTern_Lake-Otter_PAD | 0.32601 | 0.1068 | 335 | 3.052 | 0.0611 |
| CommonTern_Lake-Shiner_Lake | 0.23101 | 0.1068 | 335 | 2.163 | 0.4327 |
| CommonTern_Lake-Shiner_River | 0.25434 | 0.0807 | 335 | 3.15 | **0.0461** |
| CommonTern_Lake-Walleye_Lake | 0.24101 | 0.0885 | 335 | 2.725 | 0.1432 |
| CommonTern_Lake-Walleye_River | 0.24601 | 0.0494 | 335 | 4.975 | **<.0001** |
| CommonTern_PAD-HerringGull_Lake | -0.47143 | 0.0911 | 335 | -5.175 | **<.0001** |
| CommonTern_PAD-Otter_PAD | 0.34024 | 0.1123 | 335 | 3.031 | 0.0649 |
| CommonTern_PAD-Shiner_Lake | 0.24524 | 0.1123 | 335 | 2.185 | 0.4182 |
| CommonTern_PAD-Shiner_River | 0.26857 | 0.0878 | 335 | 3.058 | 0.0601 |
| CommonTern_PAD-Walleye_Lake | 0.25524 | 0.095 | 335 | 2.688 | 0.1561 |
| CommonTern_PAD-Walleye_River | 0.26024 | 0.0603 | 335 | 4.315 | **0.0007** |
| HerringGull_Lake-Otter_PAD | 0.81167 | 0.1312 | 335 | 6.186 | **<.0001** |
| HerringGull_Lake-Shiner_Lake | 0.71667 | 0.1312 | 335 | 5.462 | **<.0001** |
| HerringGull_Lake-Shiner_River | 0.74 | 0.111 | 335 | 6.666 | **<.0001** |
| HerringGull_Lake-Walleye_Lake | 0.72667 | 0.1167 | 335 | 6.225 | **<.0001** |
| HerringGull_Lake-Walleye_River | 0.73167 | 0.0908 | 335 | 8.056 | **<.0001** |
| Otter_PAD-Shiner_Lake | -0.095 | 0.1467 | 335 | -0.648 | 0.9993 |
| Otter_PAD-Shiner_River | -0.07167 | 0.1289 | 335 | -0.556 | 0.9998 |
| Otter_PAD-Walleye_Lake | -0.085 | 0.1339 | 335 | -0.635 | 0.9994 |
| Otter_PAD-Walleye_River | -0.08 | 0.112 | 335 | -0.714 | 0.9986 |
| Shiner_Lake-Shiner_River | 0.02333 | 0.1289 | 335 | 0.181 | 1 |
| Shiner_Lake-Walleye_Lake | 0.01 | 0.1339 | 335 | 0.075 | 1 |
| Shiner_Lake-Walleye_River | 0.015 | 0.112 | 335 | 0.134 | 1 |
| Shiner_River-Walleye_Lake | -0.01333 | 0.1142 | 335 | -0.117 | 1 |
| Shiner_River-Walleye_River | -0.00833 | 0.0875 | 335 | -0.095 | 1 |
| Walleye_Lake-Walleye_River | 0.005 | 0.0947 | 335 | 0.053 | 1 |
| **Δ^199^Hg** |  |  |  |  |  |
| **contrast** | **estimate** | **SE** | **df** | **t.ratio** | **p.value** |
| CaspianTern_Lake-CommonTern_Lake | -0.8373 | 0.0609 | 335 | -13.746 | **<.0001** |
| CaspianTern_Lake-CommonTern_PAD | -0.2736 | 0.0871 | 335 | -3.141 | **0.0474** |
| CaspianTern_Lake-HerringGull_Lake | -2.9181 | 0.1503 | 335 | -19.419 | **<.0001** |
| CaspianTern_Lake-Otter_PAD | 0.4539 | 0.1912 | 335 | 2.373 | 0.3023 |
| CaspianTern_Lake-Shiner_Lake | -0.7728 | 0.1912 | 335 | -4.041 | **0.0021** |
| CaspianTern_Lake-Shiner_River | 0.4639 | 0.1438 | 335 | 3.226 | **0.0367** |
| CaspianTern_Lake-Walleye_Lake | -0.2317 | 0.1578 | 335 | -1.468 | 0.8696 |
| CaspianTern_Lake-Walleye_River | 0.5639 | 0.0861 | 335 | 6.545 | **<.0001** |
| CommonTern_Lake-CommonTern_PAD | 0.5638 | 0.0901 | 335 | 6.257 | **<.0001** |
| CommonTern_Lake-HerringGull_Lake | -2.0808 | 0.152 | 335 | -13.687 | **<.0001** |
| CommonTern_Lake-Otter_PAD | 1.2912 | 0.1926 | 335 | 6.703 | **<.0001** |
| CommonTern_Lake-Shiner_Lake | 0.0645 | 0.1926 | 335 | 0.335 | 1 |
| CommonTern_Lake-Shiner_River | 1.3012 | 0.1456 | 335 | 8.936 | **<.0001** |
| CommonTern_Lake-Walleye_Lake | 0.6057 | 0.1595 | 335 | 3.797 | **0.0054** |
| CommonTern_Lake-Walleye_River | 1.4012 | 0.0892 | 335 | 15.714 | **<.0001** |
| CommonTern_PAD-HerringGull_Lake | -2.6446 | 0.1643 | 335 | -16.098 | **<.0001** |
| CommonTern_PAD-Otter_PAD | 0.7274 | 0.2024 | 335 | 3.593 | **0.0112** |
| CommonTern_PAD-Shiner_Lake | -0.4992 | 0.2024 | 335 | -2.466 | 0.2525 |
| CommonTern_PAD-Shiner_River | 0.7374 | 0.1584 | 335 | 4.656 | **0.0002** |
| CommonTern_PAD-Walleye_Lake | 0.0419 | 0.1712 | 335 | 0.245 | 1 |
| CommonTern_PAD-Walleye_River | 0.8374 | 0.1088 | 335 | 7.7 | **<.0001** |
| HerringGull_Lake-Otter_PAD | 3.372 | 0.2366 | 335 | 14.252 | **<.0001** |
| HerringGull_Lake-Shiner_Lake | 2.1453 | 0.2366 | 335 | 9.068 | **<.0001** |
| HerringGull_Lake-Shiner_River | 3.382 | 0.2002 | 335 | 16.894 | **<.0001** |
| HerringGull_Lake-Walleye_Lake | 2.6864 | 0.2105 | 335 | 12.761 | **<.0001** |
| HerringGull_Lake-Walleye_River | 3.482 | 0.1638 | 335 | 21.261 | **<.0001** |
| Otter_PAD-Shiner_Lake | -1.2267 | 0.2645 | 335 | -4.637 | **0.0002** |
| Otter_PAD-Shiner_River | 0.01 | 0.2325 | 335 | 0.043 | 1 |
| Otter_PAD-Walleye_Lake | -0.6856 | 0.2415 | 335 | -2.839 | 0.1081 |
| Otter_PAD-Walleye_River | 0.11 | 0.202 | 335 | 0.544 | 0.9998 |
| Shiner_Lake-Shiner_River | 1.2367 | 0.2325 | 335 | 5.318 | **<.0001** |
| Shiner_Lake-Walleye_Lake | 0.5411 | 0.2415 | 335 | 2.241 | 0.3817 |
| Shiner_Lake-Walleye_River | 1.3367 | 0.202 | 335 | 6.616 | **<.0001** |
| Shiner_River-Walleye_Lake | -0.6956 | 0.2059 | 335 | -3.378 | **0.0229** |
| Shiner_River-Walleye_River | 0.1 | 0.1578 | 335 | 0.634 | 0.9994 |
| Walleye_Lake-Walleye_River | 0.7956 | 0.1707 | 335 | 4.659 | **0.0002** |

| **Δ^200^Hg** |  |  |  |  |  |
| --- | --- | --- | --- | --- | --- |
| **contrast** | **estimate** | **SE** | **df** | **t.ratio** | **p.value** |
| CaspianTern_Lake-CommonTern_Lake | 0.004697 | 0.00616 | 335 | 0.762 | 0.9977 |
| CaspianTern_Lake-CommonTern_PAD | 0.006429 | 0.00881 | 335 | 0.73 | 0.9983 |
| CaspianTern_Lake-HerringGull_Lake | -0.04 | 0.0152 | 335 | -2.631 | 0.1777 |
| CaspianTern_Lake-Otter_PAD | 0.021667 | 0.01935 | 335 | 1.12 | 0.971 |
| CaspianTern_Lake-Shiner_Lake | 0.005 | 0.01935 | 335 | 0.258 | 1 |
| CaspianTern_Lake-Shiner_River | 0.032273 | 0.01454 | 335 | 2.219 | 0.3958 |
| CaspianTern_Lake-Walleye_Lake | 0.026111 | 0.01597 | 335 | 1.635 | 0.7847 |
| CaspianTern_Lake-Walleye_River | 0.030556 | 0.00871 | 335 | 3.506 | **0.015** |
| CommonTern_Lake-CommonTern_PAD | 0.001732 | 0.00911 | 335 | 0.19 | 1 |
| CommonTern_Lake-HerringGull_Lake | -0.0447 | 0.01538 | 335 | -2.906 | 0.0908 |
| CommonTern_Lake-Otter_PAD | 0.01697 | 0.01949 | 335 | 0.871 | 0.9943 |
| CommonTern_Lake-Shiner_Lake | 0.000303 | 0.01949 | 335 | 0.016 | 1 |
| CommonTern_Lake-Shiner_River | 0.027576 | 0.01473 | 335 | 1.872 | 0.6335 |
| CommonTern_Lake-Walleye_Lake | 0.021414 | 0.01614 | 335 | 1.327 | 0.9227 |
| CommonTern_Lake-Walleye_River | 0.025859 | 0.00902 | 335 | 2.867 | 0.1007 |
| CommonTern_PAD-HerringGull_Lake | -0.04643 | 0.01662 | 335 | -2.794 | 0.1211 |
| CommonTern_PAD-Otter_PAD | 0.015238 | 0.02048 | 335 | 0.744 | 0.9981 |
| CommonTern_PAD-Shiner_Lake | -0.00143 | 0.02048 | 335 | -0.07 | 1 |
| CommonTern_PAD-Shiner_River | 0.025844 | 0.01602 | 335 | 1.613 | 0.7971 |
| CommonTern_PAD-Walleye_Lake | 0.019683 | 0.01732 | 335 | 1.136 | 0.9683 |
| CommonTern_PAD-Walleye_River | 0.024127 | 0.011 | 335 | 2.193 | 0.4126 |
| HerringGull_Lake-Otter_PAD | 0.061667 | 0.02393 | 335 | 2.577 | 0.2005 |
| HerringGull_Lake-Shiner_Lake | 0.045 | 0.02393 | 335 | 1.88 | 0.6279 |
| HerringGull_Lake-Shiner_River | 0.072273 | 0.02025 | 335 | 3.569 | **0.0121** |
| HerringGull_Lake-Walleye_Lake | 0.066111 | 0.0213 | 335 | 3.105 | 0.0526 |
| HerringGull_Lake-Walleye_River | 0.070556 | 0.01657 | 335 | 4.259 | **0.0009** |
| Otter_PAD-Shiner_Lake | -0.01667 | 0.02676 | 335 | -0.623 | 0.9995 |
| Otter_PAD-Shiner_River | 0.010606 | 0.02352 | 335 | 0.451 | 1 |
| Otter_PAD-Walleye_Lake | 0.004444 | 0.02443 | 335 | 0.182 | 1 |
| Otter_PAD-Walleye_River | 0.008889 | 0.02044 | 335 | 0.435 | 1 |
| Shiner_Lake-Shiner_River | 0.027273 | 0.02352 | 335 | 1.159 | 0.9642 |
| Shiner_Lake-Walleye_Lake | 0.021111 | 0.02443 | 335 | 0.864 | 0.9946 |
| Shiner_Lake-Walleye_River | 0.025556 | 0.02044 | 335 | 1.25 | 0.9444 |
| Shiner_River-Walleye_Lake | -0.00616 | 0.02083 | 335 | -0.296 | 1 |
| Shiner_River-Walleye_River | -0.00172 | 0.01597 | 335 | -0.108 | 1 |
| Walleye_Lake-Walleye_River | 0.004444 | 0.01727 | 335 | 0.257 | 1 |

**Figure S3.** Biplot of odd mercury isotopes (Δ^201^Hg, Δ^199^Hg) to assess photochemical degradation of methylmercury in biota from Lake Athabasca, the Peace-Athabasca Delta and the Athabasca River. The linear regression equation is provided in plot.


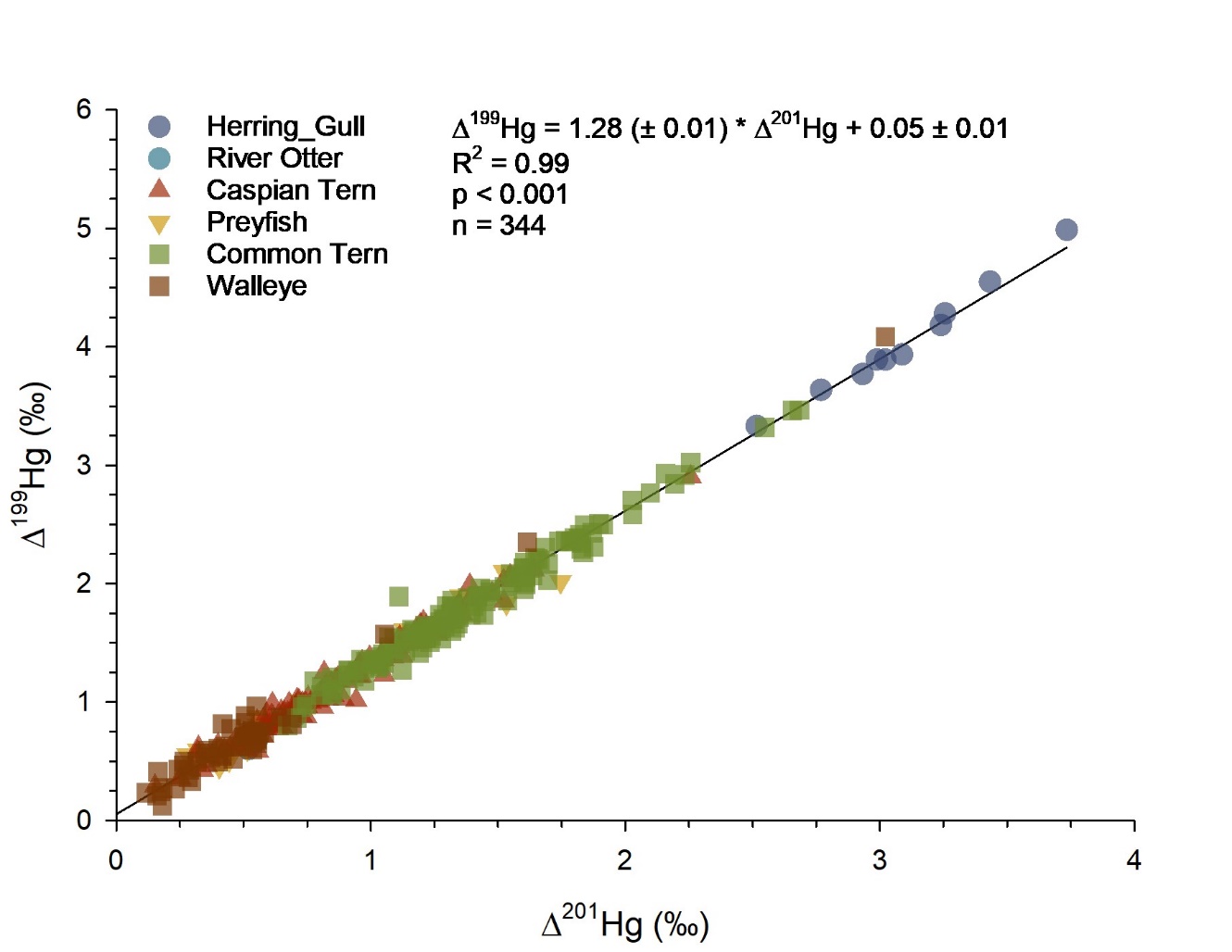


**Table S2.** Comparison of Bayesian mixing model results for estimates of river and lake contributions to mercury in biota using different combinations of Hg isotopes (δ^202^Hg, δ^202^Hg_corr_, Δ^199^Hg, Δ^200^Hg) as source tracers. MixSIAR outputs for the seven models include the proportion of mercury from the Athabasca River in biota from Lake Athabasca or the Peace-Athabasca Delta (PAD) (mean, standard deviation, 95% credible interval, size of the credible interval), the Deviance Information Criterion (DIC), the Markov chain Monte Carlo sampling length, the Gelman-Rubin Diagnostic, and the Geweke diagnostic.

| **Model** | **Tracers** | **Consumers** | **Hg Source** | **Mean Proportion** | **SD** | **2.50%** | **97.50%** | **CI Size** | **DIC** | **Source  Data Type** | **MCMC  Length** | **Gelman-Rubin Diagnostic** | **Geweke Diagnostic** |
| --- | --- | --- | --- | --- | --- | --- | --- | --- | --- | --- | --- | --- | --- |
| 1 | d202Hg | Caspian Tern - LA | River | 0.792 | 0.013 | 0.766 | 0.817 | 0.051 | 1983.102 | Mean | Long | Out of 311 variables: | Chain 1, Chain 2, Chain 3 |
|  | D199Hg | Common Tern - LA | River | 0.615 | 0.018 | 0.580 | 0.648 | 0.068 |  |  |  | 0 > 1.01 | 5, 30, 5 (out of 311) |
|  | D200Hg | Common Tern - PAD | River | 0.758 | 0.019 | 0.719 | 0.794 | 0.075 |  |  |  | 0 > 1.05 |  |
|  |  | River Otter - PAD | River | 0.957 | 0.026 | 0.893 | 0.993 | 0.100 |  |  |  | 0 > 1.1 |  |
|  |  | Preyfish - LA | River | 0.687 | 0.050 | 0.587 | 0.781 | 0.194 |  |  |  |  |  |
|  |  | Walleye - LA | River | 0.862 | 0.043 | 0.774 | 0.946 | 0.172 |  |  |  |  |  |
|  |  |  |  |  |  |  |  |  |  |  |  |  |  |
| 2 | d202Hgcorr | Caspian Tern - LA | River | 0.801 | 0.017 | 0.768 | 0.832 | 0.064 | 2163.067 | Mean | Long | Out of 311 variables: | Chain 1, Chain 2, Chain 3 |
|  | D199Hg | Common Tern - LA | River | 0.617 | 0.02 | 0.575 | 0.655 | 0.080 |  |  |  | 0 > 1.01 | 18, 17, 0 (out of 311) |
|  | D200Hg | Common Tern - PAD | River | 0.763 | 0.023 | 0.719 | 0.808 | 0.089 |  |  |  | 0 > 1.05 |  |
|  |  | River Otter - PAD | River | 0.941 | 0.035 | 0.86 | 0.991 | 0.131 |  |  |  | 0 > 1.1 |  |
|  |  | Preyfish - LA | River | 0.666 | 0.057 | 0.547 | 0.773 | 0.226 |  |  |  |  |  |
|  |  | Walleye - LA | River | 0.885 | 0.054 | 0.769 | 0.978 | 0.209 |  |  |  |  |  |
|  |  |  |  |  |  |  |  |  |  |  |  |  |  |
| 3 | d202Hgcorr | Caspian Tern - LA | River | 0.808 | 0.016 | 0.776 | 0.839 | 0.063 | 1372.536 | Mean | Long | Out of 311 variables: | Chain 1, Chain 2, Chain 3 |
|  | D199Hg | Common Tern - LA | River | 0.609 | 0.022 | 0.563 | 0.649 | 0.086 |  |  |  | 0 > 1.01 | 58, 27, 7 (out of 311) |
|  |  | Common Tern - PAD | River | 0.757 | 0.024 | 0.709 | 0.804 | 0.095 |  |  |  | 0 > 1.05 |  |
|  |  | River Otter - PAD | River | 0.948 | 0.032 | 0.875 | 0.993 | 0.118 |  |  |  | 0 > 1.1 |  |
|  |  | Preyfish - LA | River | 0.676 | 0.058 | 0.559 | 0.786 | 0.227 |  |  |  |  |  |
|  |  | Walleye - LA | River | 0.836 | 0.078 | 0.657 | 0.967 | 0.310 |  |  |  |  |  |
|  |  |  |  |  |  |  |  |  |  |  |  |  |  |
| 4 | D199Hg | Caspian Tern - LA | River | 0.836 | 0.016 | 0.804 | 0.866 | 0.062 | 1338.271 | Mean | Long | Out of 311 variables: | Chain 1, Chain 2, Chain 3 |
|  | D200Hg | Common Tern - LA | River | 0.617 | 0.021 | 0.576 | 0.657 | 0.081 |  |  |  | 0 > 1.01 | 55, 55, 10 (out of 311) |
|  |  | Common Tern - PAD | River | 0.775 | 0.025 | 0.726 | 0.823 | 0.097 |  |  |  | 0 > 1.05 |  |
|  |  | River Otter - PAD | River | 0.938 | 0.038 | 0.848 | 0.992 | 0.144 |  |  |  | 0 > 1.1 |  |
|  |  | Preyfish - LA | River | 0.638 | 0.057 | 0.522 | 0.748 | 0.226 |  |  |  |  |  |
|  |  | Walleye - LA | River | 0.853 | 0.068 | 0.71 | 0.976 | 0.266 |  |  |  |  |  |
|  |  |  |  |  |  |  |  |  |  |  |  |  |  |
| 5 | d202Hgcorr | Caspian Tern - LA | River | 0.598 | 0.053 | 0.482 | 0.691 | 0.209 | 784.320 | Mean | Long | Out of 311 variables: | Chain 1, Chain 2, Chain 3 |
|  |  | Common Tern - LA | River | 0.579 | 0.064 | 0.439 | 0.689 | 0.250 |  |  |  | 0 > 1.01 | 34, 16, 69 (out of 311) |
|  |  | Common Tern - PAD | River | 0.700 | 0.057 | 0.574 | 0.802 | 0.228 |  |  |  | 0 > 1.05 |  |
|  |  | River Otter - PAD | River | 0.907 | 0.076 | 0.71 | 0.989 | 0.279 |  |  |  | 0 > 1.1 |  |
|  |  | Preyfish - LA | River | 0.876 | 0.081 | 0.678 | 0.981 | 0.303 |  |  |  |  |  |
|  |  | Walleye - LA | River | 0.891 | 0.078 | 0.696 | 0.986 | 0.290 |  |  |  |  |  |
|  |  |  |  |  |  |  |  |  |  |  |  |  |  |
| 6 | D199Hg | Caspian Tern - LA | River | 0.841 | 0.015 | 0.811 | 0.871 | 0.060 | 540.348 | Mean | Long | Out of 311 variables: | Chain 1, Chain 2, Chain 3 |
|  |  | Common Tern - LA | River | 0.605 | 0.023 | 0.557 | 0.649 | 0.092 |  |  |  | 0 > 1.01 | 0, 13, 52 (out of 311) |
|  |  | Common Tern - PAD | River | 0.770 | 0.026 | 0.718 | 0.819 | 0.101 |  |  |  | 0 > 1.05 |  |
|  |  | River Otter - PAD | River | 0.947 | 0.034 | 0.866 | 0.993 | 0.127 |  |  |  | 0 > 1.1 |  |
|  |  | Preyfish - LA | River | 0.643 | 0.061 | 0.517 | 0.750 | 0.233 |  |  |  |  |  |
|  |  | Walleye - LA | River | 0.713 | 0.116 | 0.485 | 0.915 | 0.430 |  |  |  |  |  |
|  |  |  |  |  |  |  |  |  |  |  |  |  |  |
| 7 | D200Hg | Caspian Tern - LA | River | 0.612 | 0.108 | 0.41 | 0.832 | 0.422 | 785.396 | Mean | Long | Out of 311 variables: | Chain 1, Chain 2, Chain 3 |
|  |  | Common Tern - LA | River | 0.599 | 0.106 | 0.406 | 0.813 | 0.407 |  |  |  | 0 > 1.01 | 41, 19, 44 (out of 311) |
|  |  | Common Tern - PAD | River | 0.738 | 0.125 | 0.493 | 0.961 | 0.468 |  |  |  | 0 > 1.05 |  |
|  |  | River Otter - PAD | River | 0.648 | 0.177 | 0.305 | 0.960 | 0.655 |  |  |  | 0 > 1.1 |  |
|  |  | Preyfish - LA | River | 0.562 | 0.192 | 0.192 | 0.936 | 0.744 |  |  |  |  |  |
|  |  | Walleye - LA | River | 0.860 | 0.099 | 0.623 | 0.984 | 0.361 |  |  |  |  |  |

**Figure S4.** Endmember biplot (Δ^199^Hg, Δ^200^Hg) for the Bayesian mixing model of river and lake contributions of mercury to biota in Lake Athabasca (LA) and the Peace-Athabasca Delta (PAD). Fish from the Athabasca River and Herring Gull eggs from eastern Lake Athabasca were used to estimate the Hg isotope signatures of biologically-available mercury pools from the river (left black circle) and lake (right black circle), respectively.


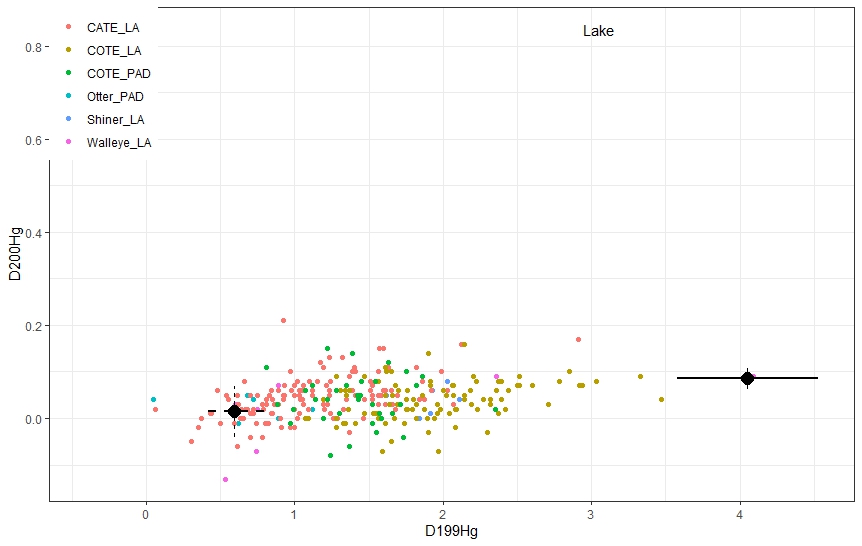


**Table S3.** Pearson coefficients for correlations between flow (m^3^/sec) of the Athabasca River or Lake Athabasca water level (m) and THg concentration (µg/g dw) of eggs of Caspian Tern and Common Tern from Lake Athabasca. Probability values for correlation coefficients were adjusted for the experiment-wide error rate using Holm’s adjustment.

| **Variable** | **Caspian Tern THg**  **(Lake Athabasca)**  **n = 12** | **Common Tern THg**  **(Lake Athabasca)**  **n = 11** |
| --- | --- | --- |
| *Athabasca River Flow* |  |  |
| Year of sampling |  |  |
| April to May | -0.16*** | 0.00*** |
| Year before sampling |  |  |
| Mean annual | **0.84***** | 0.54*** |
| June | **0.90***** | **0.74***** |
| May to August | **0.87***** | 0.52*** |
| *Athabasca Lake Water Level* |  |  |
| Year of sampling |  |  |
| April to May | 0.55*** | **0.74***** |
| Year before sampling |  |  |
| Mean annual | **0.67***** | **0.89***** |
| Peak (June or July) | **0.82***** | **0.88***** |

** adjusted p < 0.01, * adjusted p < 0.05

**Figure S5.** Positive correlations between egg THg concentration and discharge of the Athabasca River (in June of the previous year) for Caspian Tern (Pearson r = 0.90, p < 0.001, n = 12) and Common Tern (Pearson r = 0.74, p = 0.009, n = 11) from Lake Athabasca. No correlation was found for Common Tern from the Athabasca Delta (p = 0.70, n = 7), although eggs were not collected from that site in high water years.


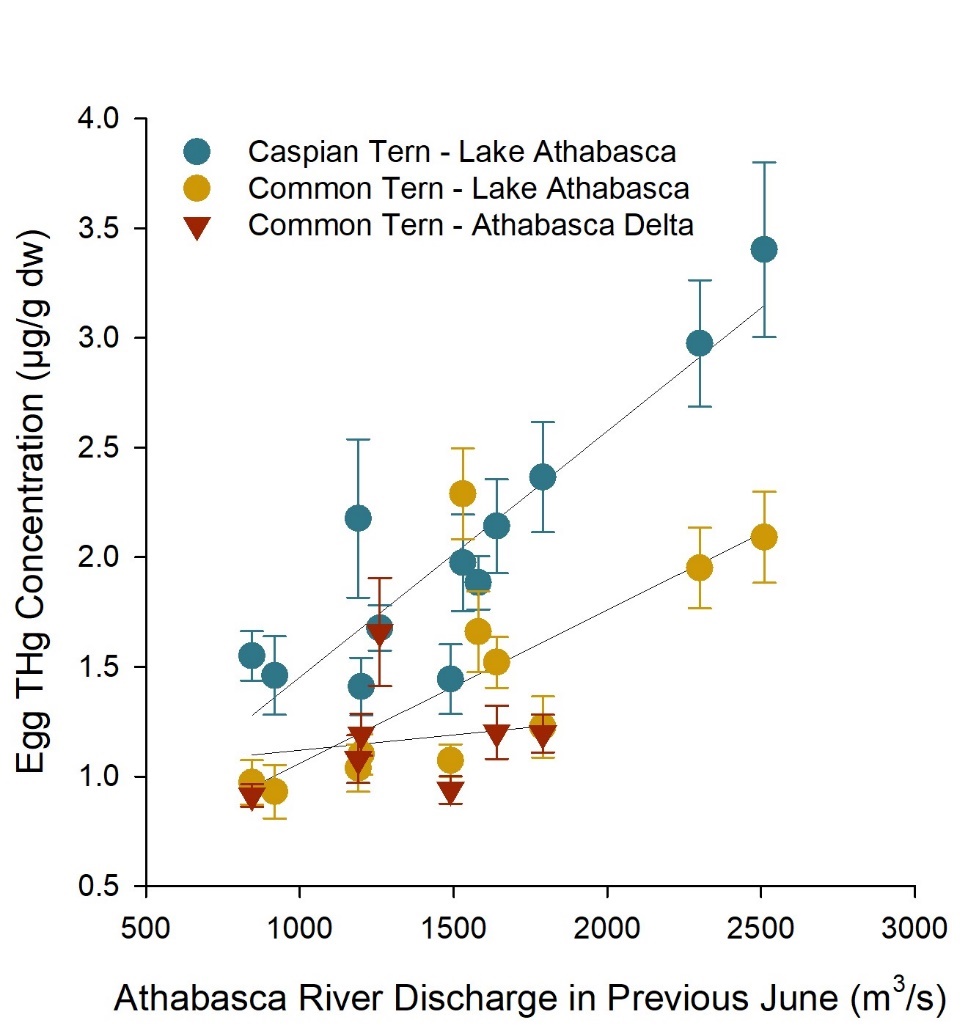


**Figure S6.** Inter-annual variation in egg THg concentration was negatively correlated with egg mercury isotope composition (Δ199Hg) for both Caspian Tern (Pearson r = -0.68, p = 0.015, n = 12) and Common Tern (Pearson r = -0.93, p < 0.001, n = 10) on Lake Athabasca. Data for Common Tern eggs from the Athabasca Delta are provided for comparison.


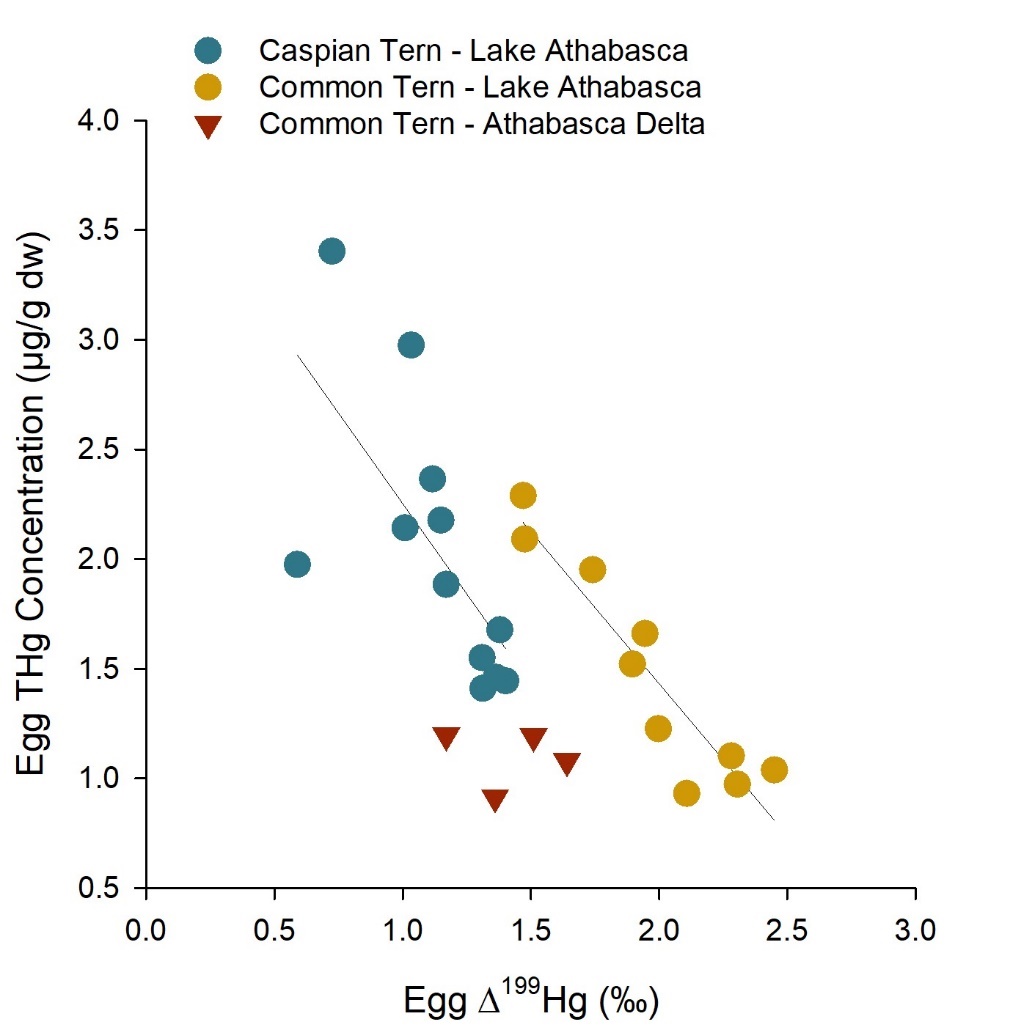


**Table S4.** Results of a linear model relating log-transformed THg concentration of Tern eggs to diet (δ^13^C [‰], δ^15^N [‰]), mercury source (δ^202^Hg_corr_ [‰], Δ^199^Hg [‰]) and hydrological variables (Athabasca River flow [m^3^/s], Athabasca Lake water level [m]). Data for Common Tern eggs from Lake Athabasca (n = 99) and the PAD (n = 35), and Caspian Tern eggs from Lake Athabasca (n = 131) are included in the model. Statistical analysis was performed in R using the “lme4” package and standardized coefficients were obtained with the effect size package.

Model: LogTHg~del202Hgcorr+D199Hg+del13C+del15N+River_Flow+Lake_Level

Model r^2^adjusted = 0.40, p < 0.001, n = 265

| **Independent Variable** | **Estimate ± SE** | **Standardized Coefficient (95% CI)** | **F-value** | **P-value** |
| --- | --- | --- | --- | --- |
| δ^202^Hg_corr_ | -0.013 ± 0.036 | -0.02 (-0.11, 0.08) | 2.7 | 0.101 |
| Δ^199^Hg | -0.073 ± 0.017 | -0.22 (-0.32, -0.12) | 77.8 | **<0.001** |
| δ^13^C | -0.014 ± 0.053 | -0.14 (-0.25, -0.03) | 25.6 | **<0.001** |
| δ^15^N | 0.039 ± 0.012 | 0.16 (0.06, 0.26) | 15.7 | **<0.001** |
| River flow^a^ | 0.001 ± 0.0004 | 0.31 (0.13, 0.49) | 60.2 | **<0.001** |
| Lake water level^b^ | 0.041 ± 0.031 | 0.13 (-0.07, 0.32) | 1.7 | 0.198 |

^a^ Athabasca River flows are means for June in the year before egg collection in accordance with correlation analysis results (Table S2).

^b^ Athabasca Lake water levels are means of peak level (in June or July) in the year before egg collection in accordance with correlation analysis results (Table S2).

**Figure S7.** Mean surface water concentrations of total mercury, dissolved mercury and methylmercury in the Athabasca River at two sampling locations. Data were obtained from the Government of Alberta Water Quality Portal (<https://environment.extranet.gov.ab.ca/apps/WaterQuality/dataportal/>) and averaged by month for all sampling years (see ranges below each figure). The top panel was for station AB07BE0010 (Athabasca River, at Town of Athabasca) and the bottom panel was for station AB07DA0980 (Athabasca River, Transect Above the Firebag River).

**Figure S8.** Longitudinal variation of methylmercury concentration in plankton of Lake Athabasca with distance from the Athabasca Delta. Data points are values of individual samples or means (± 1 standard deviation) of duplicates.


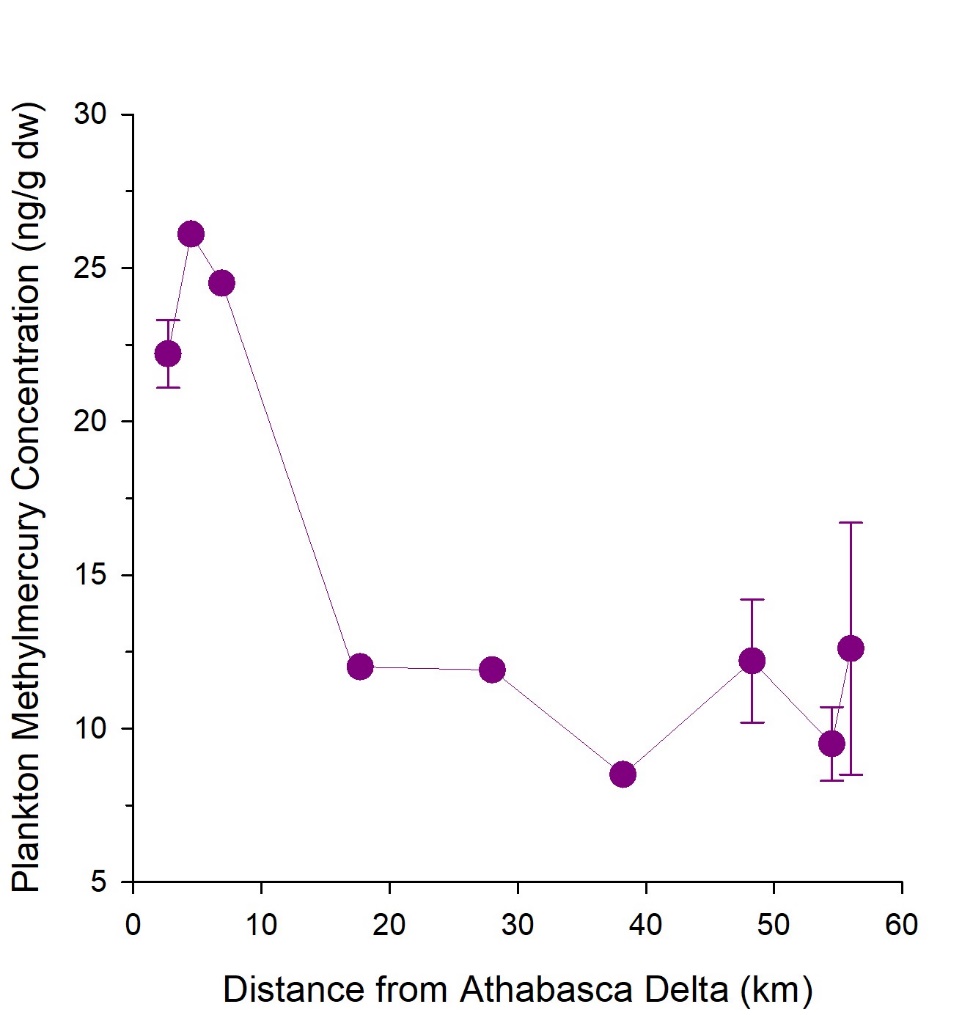


**Table S5.** Mercury concentrations and mercury stable isotope values of abiotic matrices from the Athabasca Oil Sands Region. Values are means (± 1 standard deviation) with the minimum and maximum in parentheses.

| **Sample Type** | **Year** | **N** | **[THg]**  **(ng/g dw)** | **δ^202^Hg**  **(‰)** | **Δ^199^Hg**  **(‰)** | **Δ^200^Hg**  **(‰)** |
| --- | --- | --- | --- | --- | --- | --- |
| Air (Hg0) | 2023 | 4 | 0.95 ± 0.20 ^a^  (0.78, 1.20) | 0.45 ± 0.23  (0.23, 0.77) | -0.24 ± 0.02  (-0.27, -0.22) | -0.07 ± 0.01  (-0.08, -0.06) |
| Athabasca River Sediment | 1989-92, 2021 | 10 | 25.2 ± 20.5  (7.7, 69.8) | -1.25 ± 0.12  (-1.42, -0.95) | -0.08 ± 0.05  (-0.14, 0) | 0 ± 0.01  (-0.01, 0.04) |
| Sunwapta River Sediment^b^ | 2021 | 1 | 10.6 | -0.68 | -0.18 | 0.05 |
| Bitumen Seeps | 2021 | 9 | 8.1 ± 4.4  (2.5, 15.8) | -1.85 ± 0.22  (-2.17, -1.52) | -0.13 ± 0.10  (-0.28, -0.04) | -0.01 ± 0.03  (-0.05, 0.03) |
| Lake Athabasca Sediment | 2023 | 13 | 66.1 ± 17.4  (45.4, 117.7) | -1.43 ± 0.26  (-1.92, -1.10) | 0.08 ± 0.08  (-0.07, 0.20) | 0.03 ± 0.04  (-0.05, 0.12) |
| Industry Process Samples | | | | | | |
| Petcoke | 2016 | 1 | 5.7 | -2.42 | -0.14 | -0.03 |
| Unprocessed Oil Sand | 2016 | 2 | 7.1 ± 4.2  (4.1, 10.1) | -1.78 ± 0.19  (-1.91, -1.64) | -0.34 ± 0.06  (-0.39, -0.30) | -0.02 ± 0.02  (-0.04, 0) |
| Processed Bitumen | 2016 | 1 | 7.0 | -1.36 | -0.36 | -0.03 |

^a^ Hg(0) concentration in ng/m^3^

^b^ Tributary of the Athabasca River (upstream of the Athabasca Oil Sands Region), sediment contains glacial till

**Table S6.** Percent methylmercury (MeHg) content of biotic tissues examined in this study. Values are means (± 1 standard deviation) with the minimum and maximum in parentheses.

| **Biota** | **Tissue** | **Percent MeHg** | **Source** |
| --- | --- | --- | --- |
| Walleye | Muscle | 87 ± 11  (71-107) | This study (n = 16) |
| Preyfish (Shiners) | Whole body | 96 ± 7  (85-108) | This study (n = 11) |
| Bird | Egg | 96 ± 8  (82-111) | Ackerman et al. 2013 |
| River Otter | Muscle | 92  (71-125) | Strom 2008 |

**References:**

Joshua T. Ackerman, Mark P. Herzog, and Steven E. Schwarzbach. *Environmental Science & Technology* **2013** *47* (4), 2052-2060 DOI: 10.1021/es304385y

Strom, S.M. Total Mercury and Methylmercury Residues in River Otters (*Lutra canadensis*) from Wisconsin. *Arch Environ Contam Toxicol* **54**, 546–554 (2008). https://doi.org/10.1007/s00244-007-9053-x
